# Modified Bis-pyrimidine Clamps for Triplex Formation and Their Use in SARS-CoV‑2 Detection

**DOI:** 10.1021/acsomega.5c02155

**Published:** 2025-05-23

**Authors:** Arnau Domínguez, Raimundo Gargallo, Carlos Cuestas-Ayllón, Irene Gomez-Pinto, Carme Fàbrega, Jesús Martínez de la Fuente, Masad J. Damha, Carlos González, Ramon Eritja, Anna Aviñó

**Affiliations:** † 203230 Instituto de Quimica Avanzada de Cataluña (IQAC), Consejo Superior de Investigaciones Científicas (CSIC), Jordi Girona 18-26, 08034 Barcelona, Spain; ‡ Centro de Investigación Biomédica en Red de Bioingeniería, Biomateriales y Nanomedicina (CIBER-BBN), 28029 Madrid, Spain; § Department of Inorganic and Organic Chemistry, 16724 University of Barcelona (UB), 08028 Barcelona, Spain; ∥ Department of Chemical Engineering and Analytical Chemistry, University of Barcelona (UB), 08028 Barcelona, Spain; ⊥ 82976 Instituto de Nanociencia y Materiales de Aragón (INMA), Consejo Superior de Investigaciones Científicas (CSIC), 50018 Zaragoza, Spain; # 69568 Instituto de Química Física Blas Cabrera, CSIC, Serrano 119, 28006 Madrid, Spain; ∇ Department of Chemistry, 5620 McGill University, Montreal, Quebec H3A 0B8, Canada

## Abstract

The formation of nucleic acid triple helices (“triplexes”) is an area of great interest due to their potential role in the natural and artificial regulation of gene expression or for use in analytical, diagnostic, or synthetic methods. During the coronavirus pandemic, a large search for novel methods for the detection of SARS-CoV-2 was undertaken. Based on triplex affinity capture and using polypurine reverse-Hoogsteen hairpins, a method known as Triplex Enhanced Nucleic Acid Detection Assay (TENADA) was developed for the rapid detection of SARS-CoV-2 without the need for polymerase chain reaction (PCR) amplification. In this work, to expand the targeting scope of this method, we explored triplex-forming bis-pyrimidine clamps targeting a polypurine sequence in the ORF1a region of SARS-CoV-2. To enhance parallel triplex stability, 2′-sugar and 5-methylpyrimidine modifications were incorporated into both strands of the clamps, and their effect on the triplexes formed was assessed via NMR and other biophysical methods. The results revealed distinct stabilizing effects of the modifications, influenced by their size, sugar puckering, and capacity to form short contacts with neighboring residues. The dual ability of clamps to simultaneously form Watson–Crick and Hoogsteen hydrogen bonds offers a novel perspective on the effect of modifications on triplex stability, previously unexplored with triplex-forming oligonucleotides (TFOs). Finally, the bis-pyrimidine clamps that formed the most stable parallel triplexes were applied in a thermal lateral flow (TLF) sensing device, demonstrating their potential as biosensing probes. These clamps effectively detected the synthetic DNA target with limits of detection (LoDs) ranging from 0.05 to 0.001 nM. Understanding the best modification strategies and their impact on the triplex structure will advance the development of clamps as biosensing and therapeutic agents.

## Graphical abstract

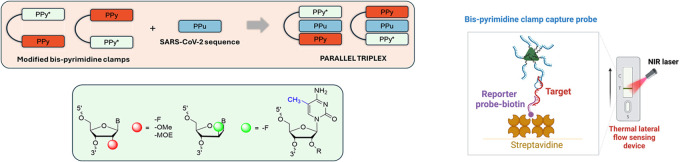

## 1 Introduction

The COVID-19 pandemic has triggered a large interest for the development of biosensing devices for rapid detection of the viral genome.^1,2^ The standard method for SARS-CoV-2 diagnosis is currently based on the quantitative reverse transcriptase polymerase chain reaction (qRT-PCR).^3^ This method needs expensive reagents and equipment and well-trained personnel and takes a few hours to be completed. The search for faster solutions has led to the development of immunological assays based on antibodies that recognize viral proteins, which are faster and do not require any special equipment. In addition, several novel detection methods have been developed, such as the use of CRISPR for signal amplification (Sherlock)^4^ and loop-mediated isothermal amplification (LAMP),^5^ together with highly selective and sensitive electrochemical biosensors.^6^

In this context, we have recently described the Triplex Enhanced Nucleic Acid Detection Assay (TENADA),^7^ a method that enables the direct detection of SARS-CoV-2 RNA using a sandwich hybridization system similar to antigen tests. TENADA takes advantage of triplex DNA structures, which have been extensively studied for “antigene” inhibition of gene expression, mutagenesis, DNA repair, as well as for specific DNA isolation by triplex affinity capture.^8–10^ Triplex formation can be achieved by the interaction of a single-stranded triplex forming oligonucleotide (TFO) with the major groove of a double-stranded DNA carrying a homopurine-homopyrimidine track, forming Hoogsteen or reverse-Hoogsteen hydrogen bonds (Figure 1a). Depending on the composition and orientation of the third strand, two types of triple helices have been described: parallel and antiparallel triplexes (Figure 1b).^8–10^ A special case of triplex-forming molecules are clamps, which are hairpin-type oligonucleotides carrying both a triplex-forming motif and a Watson–Crick strand that allow the formation of highly stable triplex structures with single or double-stranded DNA or RNA targets.^11^ These clamps can be classified depending on the type of triplex formed and the composition of their strands.

**Figure 1:**
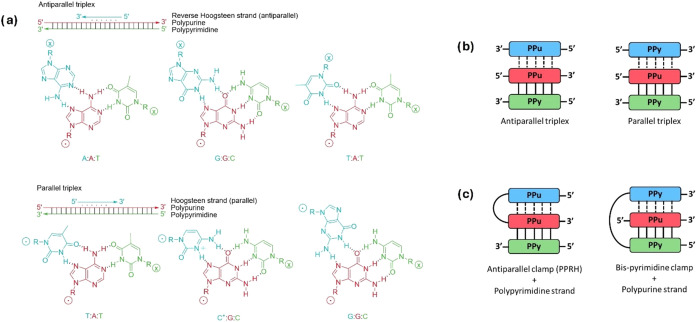
(a) Triads formed in parallel and antiparallel triplexes. Bases on the Hoogsteen or reverse-Hoogsteen strand are denoted in blue, on the Watson–Crick purine strand are denoted in red, and on the Watson–Crick pyrimidine strand are denoted in green. Representation of the triplex formed by (b) a single-stranded TFO or (c) a clamp targeting a polypyrimidine or polypurine strand. Watson–Crick hydrogen bonds are represented in solid lines, while Hoogsteen or reverse-Hoogsteen hydrogen bonds are represented in dashed lines. Pyrimidine Watson–Crick, purine Watson–Crick, and Hoogsteen or reverse-Hoogsteen strands are represented in green, red, and blue boxes, respectively.

The TENADA method initially relied on antiparallel triplex formation using antiparallel clamps, also known as polypurine reverse-Hoogsteen hairpins (PPRHs), formed by two polypurine strands linked via a tetrathymidine loop (Figure 1c). These clamps recognize polypyrimidine sequences, allowing efficient capture of the viral genome.^11^ The limit of detection (LoD) obtained using the aforementioned clamps was around 0.01 nM without the need for any amplification step.^7^ Our previous studies further characterized the biophysical properties of several PPRH targeting the longest polypyrimidine sites of SARS-CoV-2.^12^ Allowing for a maximum of three interruptions in the polypyrimidine track, we found five potential sites with a length of 20–21 nucleotides. The binding affinity of the PPRHs targeting the polypyrimidine sequences was measured and explained in terms of length, GC content, as well as the number and relative position of the purine interruptions on the polypyrimidine track.^7^ These studies also revealed a strong correlation between triplex stability and biosensor performance.^12^

During our wide analysis of potential triplex-forming sites in the SARS-CoV-2 genome, we also identified long polypurine tracks.^7^ Allowing for a maximum of three interruptions in the polypurine track, we found a potential site for triplex formation with a length of 24 nucleotides (21 purines and 3 pyrimidine interruptions). This type of polypurine sequence can also be targeted by another clamp named bis-pyrimidine clamp,^13–16^ which has been used in several applications as shown for bis-PNA openers.^17,18^ In this case, a parallel triplex is formed by two pyrimidine strands and a target polypurine strand (Figure 1c). Unlike antiparallel triplexes, which are stable under physiological conditions, the stability of parallel triplexes is dictated by the C^+^·G-C triad (Figure 1a), which requires the protonation of cytosine in the Hoogsteen strand (where “·” refers to the Hoogsteen hydrogen bonds and “-” refers to the Watson–Crick hydrogen bonds).^19–21^ Consequently, these triplexes are stabilized under mildly acidic conditions, close to the p*K*_a_ of the protonated N3 of cytosine (≈4.2).^22–24^ This limitation has triggered the development of several modified nucleosides that are able to stabilize parallel triplexes, including 5-methyl-2′-deoxycytidine,^25,26^ 2′-O-methyl-RNA,^27^ 2′-fluoro-RNA^28^ and 2′-fluoroarabino (FANA)^28,29^ derivatives.

Herein, a synthetic DNA target with the same sequence as the homopurine target found in the SARS-CoV-2 genome was used to investigate the potential application of modified bis-pyrimidine clamps for triplex capture. NMR and UV melting experiments, circular dichroism (CD) spectroscopy, electrophoretic mobility shift assays (EMSA), and pH titrations were carried out to assess the stability of the sequences and the effect of modifications in the 2′ position of the sugar moiety as well as the effect of 5-methylation of the pyrimidines. Moreover, the modified sequences that formed the most stable parallel triplexes were used as capture probes in a thermal lateral flow (TLF) sensing device. These bis-pyrimidine clamps were able to detect up to a few picomoles of synthetic DNA targets with similar sensitivities compared to the previously studied antiparallel clamps.^7,12^

## 2 Materials and Methods

### 2.1 Synthesis of Oligonucleotides

Oligonucleotides were synthesized on an H-8 DNA/RNA synthesizer (K&A Laboratories, Germany) at a 1 μmol scale using standard phosphoramidite solid-phase protocols. The coupling times for the modified nucleotides were, MOE 300, OMe 300, FANA 600, and F-ribo 900 s.

Deprotection of the oligonucleotides was accomplished after treatment with a 32% ammonium hydroxide solution overnight and 1 h at 55 °C. When the oligonucleotides were synthesized using the Glen unysupport, the deprotection time was increased to 16 h at 55 °C.

Oligonucleotides were purified using Glen-Pack purification cartridges. After that, the sequences were characterized by RP-HPLC and MALDI-TOF spectrometry.

### 2.2 UV-Determined Melting Temperatures

UV melting experiments were performed on a JASCO V-650 spectrophotometer between 20 and 80 °C for the pH 5.0 and 6.0 samples and between 10 and 80 °C for the pH 7.0 ones. Absorbance values were measured at 260 nm with a temperature ramp of 1 °C/min and with a data acquisition every 0.5 °C.

Bis-pyrimidine clamps or complementary sequences were mixed with equimolar amounts of the polypurine target (2 μM each strand) in 100 mM phosphate buffer with 100 mM NaCl at pH ranging from 5.0 to 7.0. After mixing the oligonucleotides the samples were annealed by increasing the temperature to 85 °C for five min and then allowing to cool down overnight.

Melting temperatures were determined after fitting the transitions to a single or double sigmoidal curve using the curve fitter MATLAB (The MathWorks, USA) analysis toolbox. The uncertainty of the *T*_m_ values was calculated as the standard deviation of three independent experiments.

### 2.3 Circular Dichroism Spectra

Circular dichroism (CD) spectra were measured with a JASCO-1500 spectropolarimeter using Hellma quartz cuvettes (10 mm path length and 1000 μL total volume).

Oligonucleotide samples were dissolved in a buffer containing 100 mM sodium phosphate (pH 7.0) and 100 mM NaCl. A 1:1 mixture of the bis-pyrimidine clamp with the polypurine target was prepared for a final oligonucleotide concentration of 2 μM.

CD spectra were recorded between 200 and 320 nm at 10 °C, with a scanning speed of 100 nm·min^–1^, a response time of 4 s, a data pitch of 0.5 nm, and a bandwidth of 1 nm. Buffer was subtracted after the spectrum.

### 2.4 Polyacrylamide Gel Electrophoresis

Electrophoretic mobility shift assays (EMSA) were performed on a 12% nondenaturing acrylamide/bisacrylamide 19:1 gel supplemented with 50 mM HEPES (pH 7.2), 10 mM MgCl_2_, and 5% glycerol.

A 2 μM solution of the different bis-pyrimidine clamps and complementary sequence with and without a 2 μM solution of the polypurine target was dissolved in 20 μL of a solution containing 100 mM sodium phosphate buffer (pH 6.0) with 100 mM NaCl and 12% glycerol. Before casting the gel, the samples were incubated for 30 min at 37 °C.

Electrophoresis was performed on an electrophoretic chamber at 70 V for 16 h at 10 °C. The running buffer used was 50 mM HEPES (pH 7.2) and 10 mM MgCl_2_. Finally, the gels were stained with SYBR Gold and visualized on an Amersham Imager 680 (GelLifeSciences).

### 2.5 Spectroscopically Monitored pH Titrations

The influence of pH on the formation of parallel triplexes by the polypurine target and respective clamps was studied by means of spectroscopically monitored pH titrations. These titrations were monitored with a JASCO V-650 spectrophotometer. A UV spectrum between 220 and 320 nm was recorded at every pH at 15 °C. A 100 mM Robinson–Britton buffer was used to maintain the buffer capacity linearly over the titration.^30^ A 100 mM NaCl was added to the buffer to mimic the ionic strength used for the other experiments.

To perform the titration, 5 μL of a 1 M solution of NaOH was successively added to a 2 μM solution of the 1:1 mixture of the bis-pyrimidine clamp with the polypurine target to increase the pH between 3.5 and 9.0. The pH was measured directly into the cuvette using a semimicro combination pH electrode (Thermo Scientific).

### 2.6 Multivariate Analysis

The mathematical analysis of data recorded along spectroscopically monitored pH titrations was performed using the multivariate curve resolution with alternating least-squares (MCR-ALS) toolbox for MATLAB.^31–33^ First, the spectra measured along the titrations were arranged in matrix *D*. Then, the matrix *D* was decomposed according to Lambert–Beer–Bouguer’s law following eq 1.

1 $$D=CS^{T}+E$$

In this equation, *C* corresponds to the matrix representing the fraction of each of the species present during the pH titration, *S* ^T^ is the matrix that contains the pure spectra of all the considered species, and *E* represents the data not explained by the model, which should be similar to the experimental uncertainty.

### 2.7 ^1^H NMR Experiments

Samples for NMR experiments were dissolved in 9:1 H_2_O/D_2_O mixture, in a buffer containing 100 mM sodium phosphate and 100 mM sodium chloride at pH 5.0, 6.0, or 7.0, with an oligonucleotide concentration of 100 μM. The annealing was performed by heating the samples to 90 °C for 5 min and then cooling them down overnight at room temperature.

The ^1^H NMR spectra were recorded at 5 °C on a Bruker Avance spectrometer equipped with a cryoprobe and operating at 600 MHz. For the melting experiments, five independent measurements were performed at 10 °C intervals, ranging from 5 and 45 °C. The data was processed using Bruker Topspin software.

### 2.8 Thermal Lateral Flow Sensing Device

To conduct the thermal lateral flow experiments, a new set of oligonucleotides was designed and synthesized following previously reported methods (Table S3).^7,12^ The first oligonucleotides were the selected modified bis-pyrimidine clamps functionalized at their 5′ end with a tetrathymidine linker and an aminohexyl group. These oligonucleotides were then immobilized onto the surface of gold nanoprisms for use as capture probes (Figure 9). Additionally, a second oligonucleotide, termed reporter probe, was synthesized. This oligonucleotide binds to a region near the binding site of the capture probe and is labeled with a biotin molecule at its 3′ end to enable interaction with the surface of the lateral flow strip. Finally, a longer synthetic DNA target (LongTarget) was synthesized with an additional binding region for the reporter probe. This LongTarget is a DNA oligonucleotide that is used to assess the suitability of the clamps for their used in this thermal lateral flow assay. The sequences used in the lateral flow experiments are given in Table S3.

For the determination of the limit of detection (LOD), 25 μL of different spiked sample solutions containing increasing amounts of the DNA target (LongTarget) sequence, ranging from 0 to 0.5 pmol, were prepared. Each sample was then combined with 10 μg of biofunctionalized nanoprisms, 0.0638 nmol (26.6 μL) of a biotinylated reporter probe, and 23.4 μL of running buffer. The resulting mixture was preincubated at room temperature for 15 min to allow optimal interaction between components. Following the incubation, 100 μL of the solution was loaded into the cassette of the lateral flow (LF) test strip. The test was run for 15 min, after which the strips were dried at 37 °C for an additional 15 min to ensure proper stabilization. For signal development, the dried strips were exposed to a near-infrared (NIR) laser (1064 nm, 1250 mW) for 1 min. Upon laser irradiation, samples with high concentrations of the DNA target sequence produced a dark brown spot on the nitrocellulose membrane of the test strip as well as on the thermosensitive paper located on the backside of the strip. In contrast, samples with lower DNA target concentrations generated a detectable signal exclusively on thermosensitive paper, indicating a reduced presence of the target sequence.

The following paragraph summarizes the procedure for LOD determination using the thermal lateral flow biosensor: (1) Addition of the sample to a solution containing Gold Nanoprisms and a biotin-labeled reporter oligonucleotide, (2) Preincubation, (3) Transfer of the preincubated sample to the PLF strip, allowing the sample to flow for 15 min, (4) Sample drying at 37 °C for 15 min, (5) Test development through NIR laser irradiation.

The signal generated on thermosensitive paper after laser irradiation can be easily quantified using a conventional lateral flow (LF) test reader. This system processes the color image by converting it into a grayscale, allowing for precise intensity measurement of the detected signal. The grayscale intensity of the spot is then quantified by the device, and the obtained data (height) are plotted against the target concentration to establish a correlation.

The “height” of the signal can be quantified by measuring the pixel values of the region where the signal appears. These pixel values represent the amount of color (grayscale) in that region, which correlates with the amount of analyte present in the sample.

## 3 Results and Discussion

### 3.1 Design and Optimization of Bis-pyrimidine Clamps

The bis-pyrimidine clamps studied in this work were designed to target a polypurine sequence within the ORF1a region of the SARS-CoV-2 viral genome (Figure 2a). Target selection was carried out using the Triplex-Forming Oligonucleotide Target Sequence Search software from the University of Texas (http://utw10685.utweb.utexas.edu/tfo/). The software output is given as the polypurine sequences that can be found on either the forward or reverse strands in the genome of interest ordered by length. The parameters used for the search were set as follows: maximum length, 30% minimum GC content, and a maximum of three pyrimidine interruptions. As SARS-CoV-2 is a single strand RNA positive virus (+ssRNA), the longest sequence found in the forward strand was chosen for the studies.^11^

**Figure 2:**
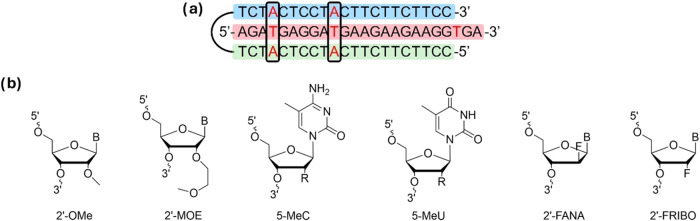
(a) Parallel triplex formed by the bis-pyrimidine clamp studied and the polypurine DNA target. Pyrimidine Watson–Crick, purine Watson–Crick, and Hoogsteen strands are represented in green, red, and blue boxes, respectively. (b) Chemical structures of the 2′-sugar and 5-methylpyrimidine modifications.

To determine the optimal design forming the most stable parallel triplex, an initial optimization of the unmodified bis-pyrimidine clamp was conducted to achieve the best balance between length and the number of pyrimidine interruptions. Three bis-pyrimidine clamps were synthesized, one with full complementarity containing three pyrimidine interruptions and two additional variants with shorter strands featuring two and one interruptions, respectively (Figure S1a). Among these, the design with two interruptions exhibited the highest stability (Table S2 and Figure S1) and was selected for subsequent studies, for now on Unmodified clamp. Additionally, a complementary sequence that can form only a duplex (Complementary) was also designed for comparison purposes (Table S1).

To further optimize stability, the selected bis-pyrimidine clamp was modified at either the Watson–Crick or Hoogsteen strand with 2′-sugar and 5-methyl-pyrimidine modifications (Figure 2b). A control sequence, named Control, with a randomized Hoogsteen sequence, which can form only a duplex, was also included. Details of the sequences studied in this work can be found in Table S1 and Figure S2.

### 3.2 Characterization and Stability of Parallel Triplexes Formed by Bis-pyrimidine Clamps Using UV, CD, EMSA, and NMR

The formation and thermal stability of the parallel triplexes formed by the studied bis-pyrimidine clamps were initially investigated by UV melting experiments performed at different pH values. All sequences exhibited thermal transitions indicative of triplex formation, with one transition decreasing as the pH increased, consistent with the pH sensitivity of parallel triplexes (Figure S3).

To further confirm the formation of the triplex structures, additional studies using circular dichroism (CD) spectroscopy were conducted for both Unmodified and 2′-sugar-modified clamps in the presence of the polypurine target at pH 5.0, 6.0, and 7.0 (Figures 3 and S4). These triplexes have a characteristic CD profile that can be easily differentiated from other structures, such as canonical double helices, G-quadruplexes, or i-motifs. At pH 5.0, both the unmodified and modified clamps displayed the typical CD profile of parallel triplexes with a minimum at 215 nm and a maximum between 260 and 280 nm.^23^ As the pH increased to 6.0, the parallel triplex signature remained visible but the signal was significantly stronger for some of the modified versions, indicating improved stabilization. At pH 7.0, while the parallel triplex profile was lost for the mixture of the Unmodified clamp with the target, some of the modified clamps still retained triplex features, demonstrating an enhanced ability to form stable parallel triplexes at neutral pH conditions (Figures 3 and S4).

**Figure 3:**
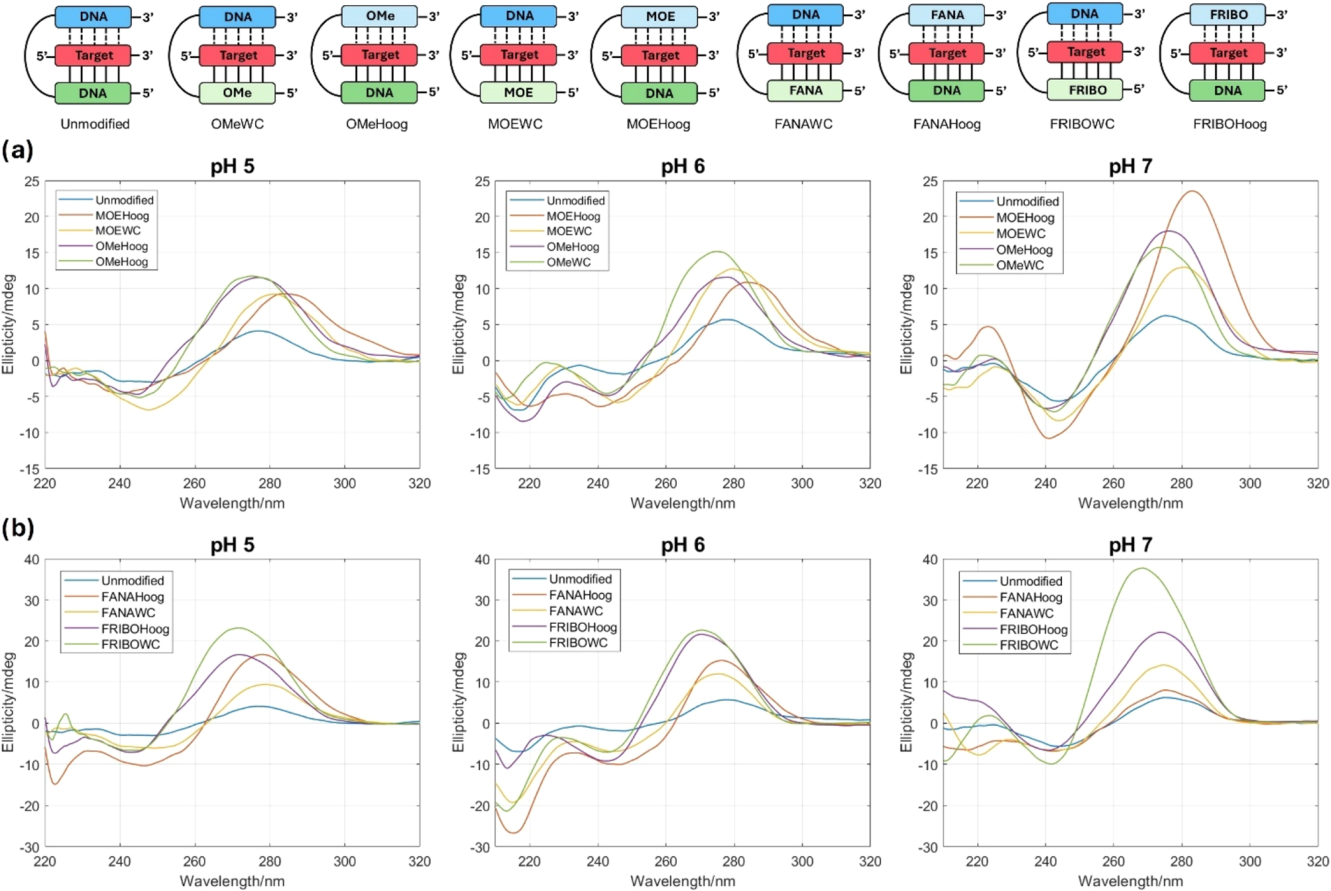
Circular dichroism spectra recorded between 320 and 200 nm of the (a) 2′-*O*-alkyl modified and (b) 2′-fluorinated clamps at pH 5.0, 6.0, and 7.0 and *T* = 10 °C. pH 5.0:100 mM sodium phosphate/citrate buffer and 100 mM NaCl. pH 6.0, 7.0:100 mM sodium phosphate and 100 mM NaCl. The oligonucleotide concentration was 2 μM. Spectra recorded at pH 5.0 between 220 and 200 nm are not shown due to buffer noise.

Electrophoretic mobility shift assays (EMSA) supported previous experiments on triplex formation, displaying a more retained band upon addition of the target to the bis-pyrimidine clamp compared to the complementary sequence. Moreover, other highly retained bands were observed for most clamps, indicating the formation of multimeric structures, although these were much less populated. The Control sequence displayed two multimeric structures with different shifts compared to the parallel triplexes formed by the bis-pyrimidine clamps (Figures 4 and S5).

**Figure 4:**
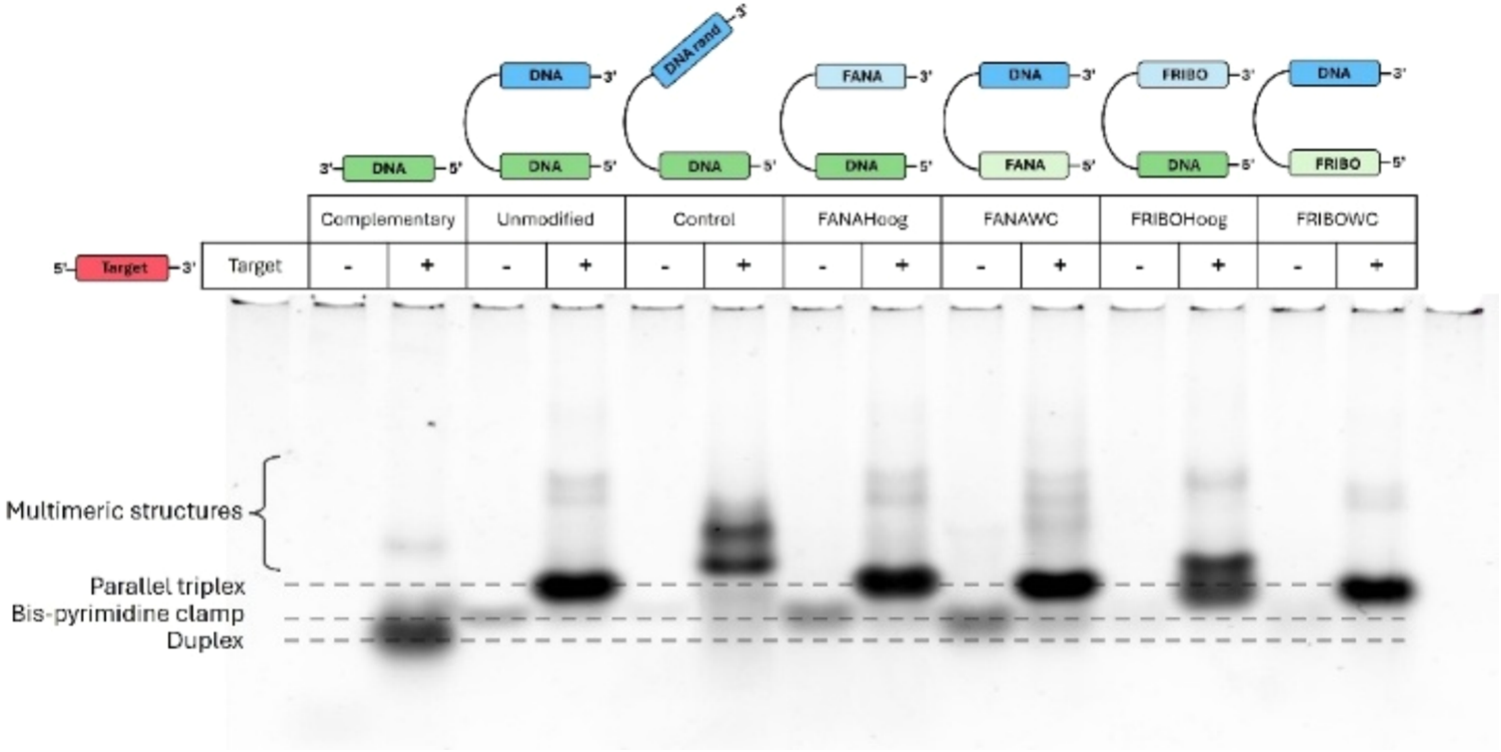
Native polyacrylamide gel electrophoresis (12%) comparing Unmodified, Complementary, Control, and 2′-fluorinated clamps with and without the addition of the polypurine target. The gels were run with 100 mM sodium phosphate buffer (pH, 6.0) and 100 mM NaCl. Gels were run at 10 °C. The oligonucleotide concentration was 2 μM.

The formation and stability of the parallel triplexes formed by the bis-pyrimidine clamps were further studied using ^1^H NMR experiments. Spectra of the 1:1 mixture of the bis-pyrimidine clamps with the polypurine target were recorded between 5 and 45 °C at pH 5.0, 6.0, and 7.0. Additionally, the same experiments were conducted with the 1:1 mixture of the Complementary sequence with the target (Figure S6). This sequence showed the characteristic imino proton signals of G:C and A:T Watson–Crick base pairs between 12.0–13.0 and 13.0–14.5 ppm, respectively. These signals were still present up to 45 °C and were not affected by pH changes, in consistency with a canonical duplex structure.^34^

The NMR spectra of the bis-pyrimidine clamps in the presence of the polypurine target showed to be highly affected by pH changes. At acidic pH, additional signals in the region between 14.5 and 16.5 ppm and between 13.0 and 14.5 ppm were observed in comparison with the Complementary sequence. These signals are consistent with protonated imino N3 of cytosines and imino protons of thymines forming Hoogsteen hydrogen bonds, present in parallel triplexes.^35^ Signals corresponding to the amino protons of protonated cytosines are also observed between 9.5 and 10.5 ppm. In some cases, these imino signals are observed at neutral pH (see Figures 5 and S6), in total agreement with the parallel triplex formation detected by CD and EMSA experiments.

**Figure 5:**
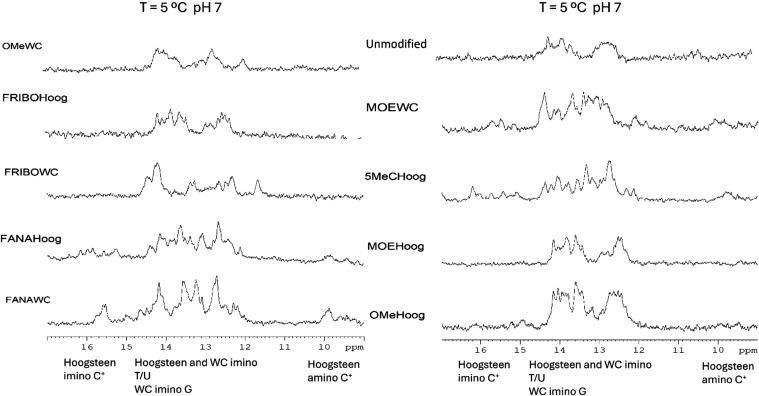
^1^H NMR spectra of the exchangeable imino region of 2′-*O*-alkyl and 2′-fluorinated clamps recorded at 5 °C. 9:1 H_2_O/D_2_O in 100 mM sodium phosphate buffer (pH, 7.0) and 100 mM NaCl. The oligonucleotide concentration was 100 μM.

### 3.3 Effect of 2′-*O*-Alkyl Modifications

The first modifications incorporated into the Watson–Crick and Hoogsteen strands were 2′-*O*-methyl (2′-OMe) and 2′-*O*-methoxyethyl (2′-MOE). Surprisingly, these two modifications exhibited opposite stabilization trends when located on different strands of the clamp (Table 1). While 2′-MOE demonstrated greater stabilization in the Watson–Crick strand (MOEWC), 2′-OMe showed a significant destabilization effect (OMeWC). In contrast, 2′-MOE destabilized the parallel triplexes in the Hoogsteen strand (MOEHoog), whereas 2′-OMe (OMeHoog) stabilized them by over 8.0 °C at pH 7.0 compared to the Unmodified clamp (Figure S3 and Table 1). These different effects are confirmed by the NMR spectra recorded at pH 7, although both 2′-OMe and 2′-MOE modifications tend to render spectra with broader imino signals (Figure 5). The stabilization effect of OMeHoog is consistent with previous works that studied TFOs targeting DNA duplexes.^27^ This stabilization effect has been attributed to a hydrophobic effect of the 2′-OMe modifications all over the Hoogsteen strand that counters the repulsion of the negatively charged phosphate groups. Conversely, although 2′-MOE residues should behave similarly to the 2′-OMe, their larger size could potentially lead to steric clashes and may not be well accommodated within the major groove of the double helix, thereby destabilizing the triplex.

**Table 1:** *T*_m_ Values Determined from the UV Melting Curves of the 1:1 Mixture of Clamps with the Polypurine Target at pH 5.0, 6.0, and 7.0 Monitored at 260 nm^a^

	*T*_m_ triplex (°C)		*T*_m_ duplex (°C)			Δ*T*_m_ triplex	
sequence	pH 6.0	pH 7.0	pH 5.0^b^	pH 6.0	pH 7.0	pH 6.0	pH 7.0
Unmodified	44.0 ± 0.4	11.4 ± 0.1	65.2 ± 0.3	64.7 ± 0.2	62.2 ± 0.2		
Complementary	n.d	n.d	56.0 ± 0.1	63.1 ± 0.1	62.1 ± 0.1	n.d	n.d
Control	n.d	n.d	56.0 ± 0.5	62.3 ± 0.6	62.9 ± 0.7	n.d	n.d
MOEHoog	40.5 ± 0.2	n.d	64.8 ± 0.3	61.7 ± 0.3	62.6 ± 0.1	–3.5	n.d
MOEWC	48.9 ± 0.6	23.5 ± 1.0	68.8 ± 0.7	71.7 ± 0.6	73.0 ± 0.2	+4.9	+12.1
OMeHoog	n.d	19.4 ± 0.1	71.3 ± 0.1	60.4 ± 0.2	61.8 ± 0.1	n.d	+8.0
OMeWC	n.d^c^	n.d^c^	55.5 ± 0.1	54.5 ± 0.3	55.9 ± 0.1	n.d	n.d
5-Me-dCWC	n.d	n.d	65.2 ± 0.2	67.6 ± 0.1	68.2 ± 1.0	n.d	n.d
5-Me-dCHoog	39.9 ± 0.5	13.9 ± 0.1	57.3 ± 0.1	58.0 ± 0.1	58.3 ± 0.1	–4.1	+2.5
FANAHoog	46.7 ± 0.3	18.1 ± 0.5	62.6 ± 0.1	61.0 ± 0.2	61.9 ± 0.1	+2.7	+6.7
FANAWC	54.4 ± 0.4	25.0 ± 0.7	69.5 ± 0.1	66.2 ± 0.2	66.6 ± 0.7	+10.4	+13.6
FRIBOHoog	26.3 ± 0.1	n.d	56.8 ± 0.4	60.7 ± 0.4	61.4 ± 0.7	–17.7	n.d
FRIBOWC	35.3 ± 0.3	n.d	62.2 ± 0.1	68.3 ± 0.1	67.9 ± 0.3	–8.7	n.d

a pH 5.0:100 mM sodium phosphate/citrate buffer and 100 mM NaCl. pH 6.0,7.0:100 mM sodium phosphate and 100 mM NaCl. The oligonucleotide concentration was 2 μM.

b Single transition observed at pH 5.0.

c pH-independent transition observed at 38.1 ± 0.3 °C. n.d: Not detected.

On the other hand, the greatest stabilization was observed when 2′-MOE modifications were positioned on the Watson–Crick strand, with an increase of 12.1 °C at pH 7.0 (MOEWC in Figure S3 and Table 1). OMeWC showed the opposite effect and did not exhibit any transition attributed to the triplex formation. Instead, two pH-independent transitions were observed at 38.1 ± 0.3 and 55.9 ± 0.1 °C. It is important to note that 2′-MOE modifications contain 5-methyl-cytidine and 5-methyl-uridine residues, while 2′-OMe contain cytidine and uridine. Thus, the stabilization effect of MOEWC could also be attributed to the 5-methyl-pyrimidines and not exclusively to the 2′-MOE residues. This topic will be discussed in the following section.

### 3.4 Effect of 5-Methyl-pyrimidine Modifications

To investigate if the presence of 5-methyl-pyrimidines in the 2′-MOE residues studied in the previous section could explain the different stabilizing trends observed for 2′-*O*-alkyl modifications, two additional clamps were synthesized, substituting cytosines with 5-methyl-cytosines in the Hoogsteen and Watson–Crick strands (5MedCHoog and 5MedCWC in Table S1). This modification has been reported to stabilize parallel triplexes at neutral pH by increasing the p*K*_a_ of the cytosines and through major groove interactions of the methyl groups when positioned on the Hoogsteen strand.^25,26,36^ UV-monitored melting and CD spectra revealed that the substitution of cytosines by 5-methyl-cytosines in the Watson–Crick strand destabilized the parallel triplexes (5MedCWC in Figure S4 and Table 1). No transition indicative of triplex formation was observed under any of the pH conditions studied.

In contrast, when 5-methyl-cytosines were positioned in the Hoogsteen strand, the melting temperature of the triplex increased by 2.5 °C, at pH 7.0 (5MedCHoog in Table 1). Triplex formation at neutral pH is clearly confirmed by the NMR spectra shown in Figure S6. This finding suggests that the stabilization observed for the MOEWC clamp is likely due to the effect of the 2′-MOE residues or a synergistic effect with the 5-methyl-pyrimidines.

### 3.5 Effect of 2′-Fluorinated Modifications

Next, 2′-fluororibo (2′-FRIBO) and 2′-fluoroarabino (2′-FANA) modifications were introduced into the bis-pyrimidine clamps. These fluorinated analogues have been demonstrated to stabilize a wide range of DNA structures, including canonical double helices and other noncanonical structures such as G-quadruplexes and i-motifs.^37–39^ In the case of 2′-FRIBO modifications, UV melting data indicate a destabilizing effect on the Watson–Crick and Hoogsteen strands (FRIBOWC and FRIBOHoog in Figure S3 and Table 1). This is confirmed by the NMR spectra shown in Figure S5. This effect could be attributed to a shift in the sugar pucker to a C3′-endo conformation, which reduces the flexibility of the structure and hampers the accommodation of a third strand in the major groove. Additionally, the absence of the 2′–OH group eliminates the possibility of forming hydrogen bonds with the phosphate groups of the purine Watson–Crick strand, which have been reported to stabilize some hybrid DNA/RNA triplexes.^27,28^ Notably, the destabilization effect was more pronounced when the 2′-FRIBO modifications were positioned on the Hoogsteen strand (FRIBOHoog), possibly due to a decreased basicity of cytosines induced by the presence of the fluorine atom at the 2′-position of the ribose.^27^

In contrast, 2′-FANA modifications demonstrated a significant stabilizing effect when positioned on both Watson–Crick and Hoogsteen strands under all pH conditions studied (FANAWC and FANAHoog in Figure S3 and Table 1). The NMR spectra of these oligonucleotides exhibit sharp and highly dispersed imino proton signals even at neutral conditions (Figures 5, 6, and S6). Temperature and pH behavior of these signals clearly indicate the formation of highly stable structures. Most probably, the preferences of 2′-FANA sugar moiety for the C2′-endo conformation favor the arrangement of the three strands to form the triplex motif.^28^ The stabilization is less pronounced when the 2′-FANA residues are located in the Hoogsteen strand. This might be due to a decreased p*K*_a_ of the 2′-FANA cytosines.

**Figure 6:**
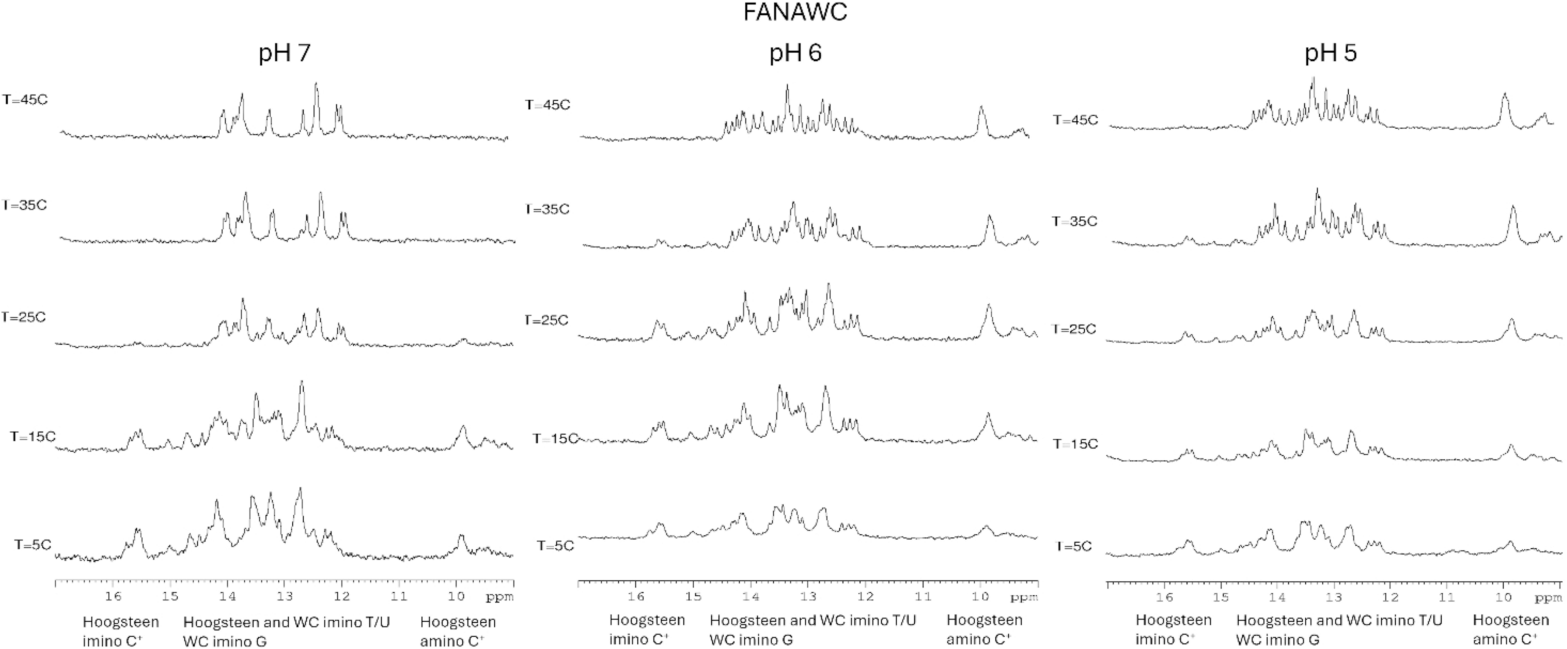
^1^H NMR melting experiments of the exchangeable imino region of FANAWC at pH 7.0, 6.0, and 5.0, and *T* = 5 °C. 9:1 H_2_O/D_2_O in 100 mM sodium phosphate buffer and 100 mM NaCl. The oligonucleotide concentration was 100 μM.

Circular dichroism spectra of the mixtures of the target with the fluorinated bis-pyrimidine clamps showed the characteristic parallel triplex profile for all the oligonucleotides at pH 5.0 and 6.0 and for the 2′-FANA modified ones at pH 7.0 (Figure 3).^23^ Interestingly, distinctive trends were observed in the region between 260 and 280 nm, where the 2′-FANA modified clamps displayed a red-shifted maximum compared to the 2′-FRIBO modifications. This observation supports the hypothesis of a C2′-endo vs C3′-endo shift in the equilibrium induced by the fluorinated modifications, as this maximum resembles the one observed in A-like (C3′-endo, blue-shifted maximum) and B-like (C2′-endo, red-shifted maximum) double helices.^40^

### 3.6 Spectroscopically Monitored pH Titrations

To obtain further insight into the major species present at neutral pH and the effect of the modifications on the acid–base properties of the parallel triplexes formed, pH-titration experiments were conducted. The variation of the spectra of the 1:1 mixtures of the Target sequence with the clamps along the titration between pH 3.5 and 9.0 is shown in Figures 7 and S8. These spectra are characterized by different absorbances at 300 nm that decrease with increasing pH. The absorbance at around 300 nm has been associated with the protonation of cytosines.^41^ Moreover, changes at around 265 nm are also predominant in the experiments with the clamps that showed the highest triplex stability and are associated with the folding of the structures.^42^ During the first transition, the absorbance decreases due to the formation of the parallel triplex, followed by a drastic increase during the second transition due to the unfolding of the Hoogsteen strand.

**Figure 7:**
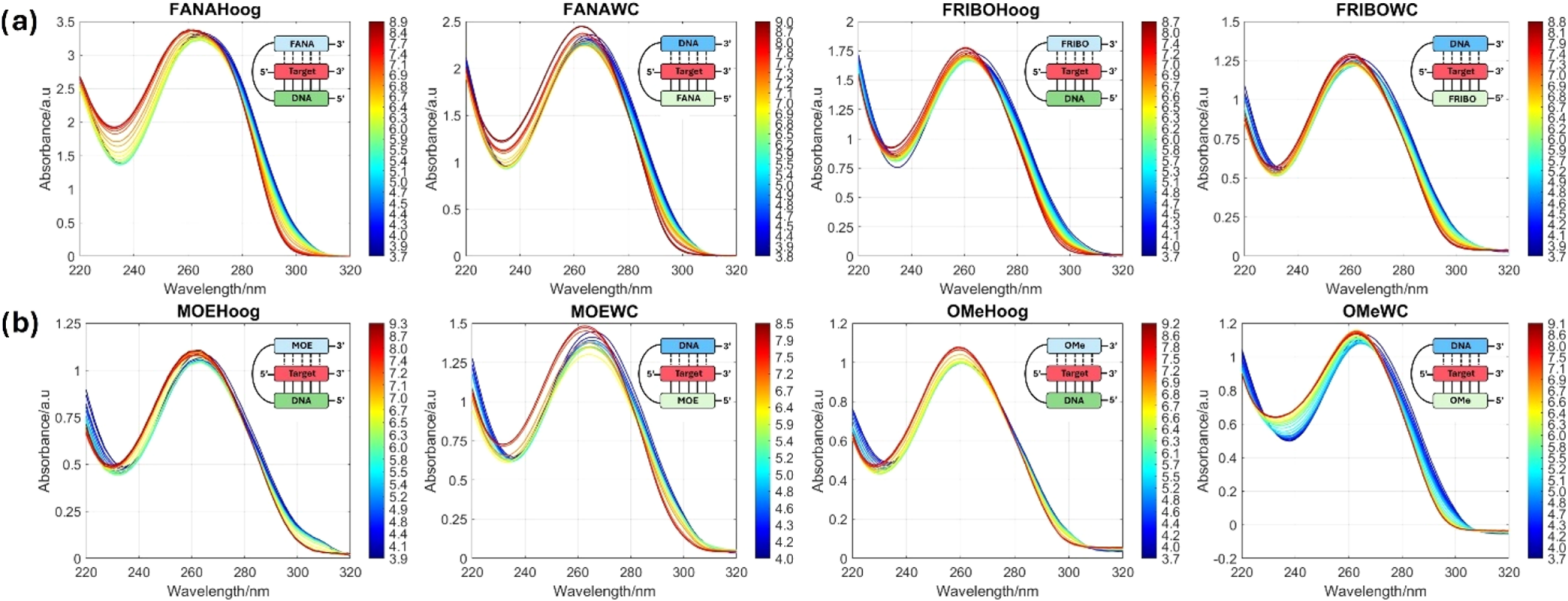
UV spectra of modified clamps recorded between 320 and 220 nm during pH titrations. (a) The 2′-fluorinated series and (b) the 2′-*O*-alkyl series. The buffer used was 100 mM Robinson–Britton with 100 mM NaCl. The oligonucleotide concentration was 2 μM.

These results were analyzed by a multivariate method using a model of three species.^23,33,43^ At pH values lower than 4.2, we expect a structure where all the cytosines are protonated and cannot form Watson–Crick hydrogen bonds.^24^ At pH values higher than 4.2, we expect the formation of the parallel triplex until the cytosines of the Hoogsteen strand are deprotonated, leading to the unwinding of the third strand at neutral pH conditions.

Most of the studied sequences exhibited a double transition, consistent with the proposed model of the three species. The first transition, observed at more acidic pH values, is associated with the direct impact of the modifications on the p*K*_a_ of cytosines and is expected to play a significant role under neutral conditions. The second transition instead, observed at more neutral pH values, can be associated with the overall stability of the structure across various pH ranges.

The highest p*K*_1/2_ of the second transition were those of the triplexes formed by the MOEWC and FANAWC clamps, with values of 7.2 ± 0.1 and 7.46 ± 0.05 respectively (Table 2, Figures S7 and S8). These clamps were also the ones that, upon interaction with the Target sequence, displayed the highest thermal stability at pH 7.0, showing a correlation with their acid–base properties. The rest of the Target:clamp mixtures had similar p*K*_1/2_ between the range of 6.4–6.9. The triplex formed by the OMeWC clamp displayed three transitions during the titration that suggested the formation of two different species in equilibrium between 5.0 and 6.5. The MCR-ALS resolved concentration profiles of the species present during the pH titration and the corresponding pure spectra are shown in Figures 8, S9, and S10. These data correlate with the experimental values shown in Figures 7 and S8.

**Figure 8:**
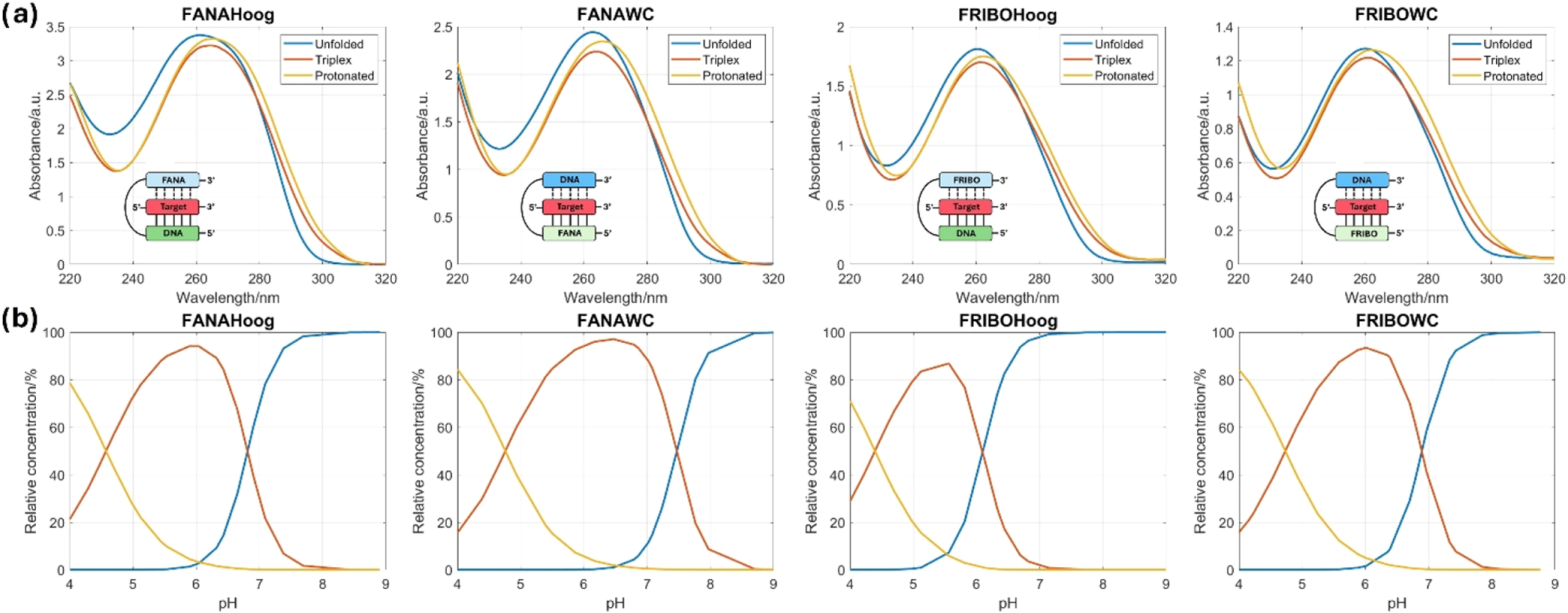
(a) Concentration profiles obtained from the multivariate analysis of the whole set of spectra recorded along the acid–base titrations of the 2′-fluorinated clamps. (b) Pure spectra of the species. Unfolded structure, parallel triplex, and duplex formed by the target and the WC strand of the clamp are represented, respectively, in yellow, red, and blue.

**Table 2:** pK_1/2_ Values for the Modified Bis-pyrimidine Clamps Determined from pH-Titration Experiments^a^

	first transition	second transition		first transition	second transition
name	p*K*_1/2_	p*K*_1/2_	name	p*K*_1/2_	p*K*_1/2_
Unmodified	4.26 ± 0.06	6.46 ± 0.05	5-Me-dCWC	3.9 ± 0.1	6.91 ± 0.05
Control	4.52 ± 0.01	5.53 ± 0.01^b^	5-Me-dCHoog	3.72 ± 0.09	6.96 ± 0.02^d^
MOEHoog	4.58 ± 0.05	6.60 ± 0.03	FANAHoog	4.57 ± 0.05	6.81 ± 0.01
MOEWC	4.74 ± 0.05	7.2 ± 0.1	FANAWC	4.76 ± 0.06	7.46 ± 0.05
OMeHoog	4.57 ± 0.04	6.89 ± 0.02	FRIBOHoog	4.4 ± 0.1	6.10 ± 0.03
OMeWC	3.8 ± 0.2	6.45 ± 0.06^c^	FRIBOWC	4.73 ± 0.05	6.89 ± 0.06

a 100 mM Robinson–Britton buffer, 100 mM NaCl, and 2 μM oligonucleotide concentration.

b Another transition at 6.6 ± 0.1 was observed.

c Another transition at 5.11 ± 0.06 was observed.

d Another transition at 5.51 ± 0.08 was observed.

### 3.7 Applicability of the Designs in a Thermal Lateral Flow Sensing Device

To demonstrate the applicability of the studied bis-pyrimidine clamps, we selected the sequences that formed the most stable parallel triplexes (2′-FANA and 2′-MOE) and tested them in a thermal lateral flow sensing device. This device utilizes the Triplex Enhanced Nucleic Acid Detection Assay (TENADA), a method previously used to detect polypyrimidine sequences within SARS-CoV-2 mRNA using antiparallel clamps.^7^

Briefly, TENADA is based on a sandwich hybridization system involving two oligonucleotides: the capture probe, which in this case consists of the selected bis-pyrimidine clamps, and the reporter probe, a single-stranded oligonucleotide labeled at its 3′-end with a biotin molecule that binds to a region near the polypurine target site. As described in Section 2.8, the capture probe is functionalized onto the surface of a gold nanoprism via an amino group. In the presence of the target sequence, a trimeric complex forms between the target, capture, and reporter probes. When the sample is loaded onto the strip, this complex is retained at the test line due to the interaction of the streptavidin-coated surface and the biotin-labeled reporter probe. Subsequently, the strip is irradiated with a laser, causing the retained gold nanoprisms to generate heat through plasmonic resonance. This localized heating results in a dark spot on the back part of the strip, indicating the presence of the target sequence (Figure 9).

**Figure 9:**
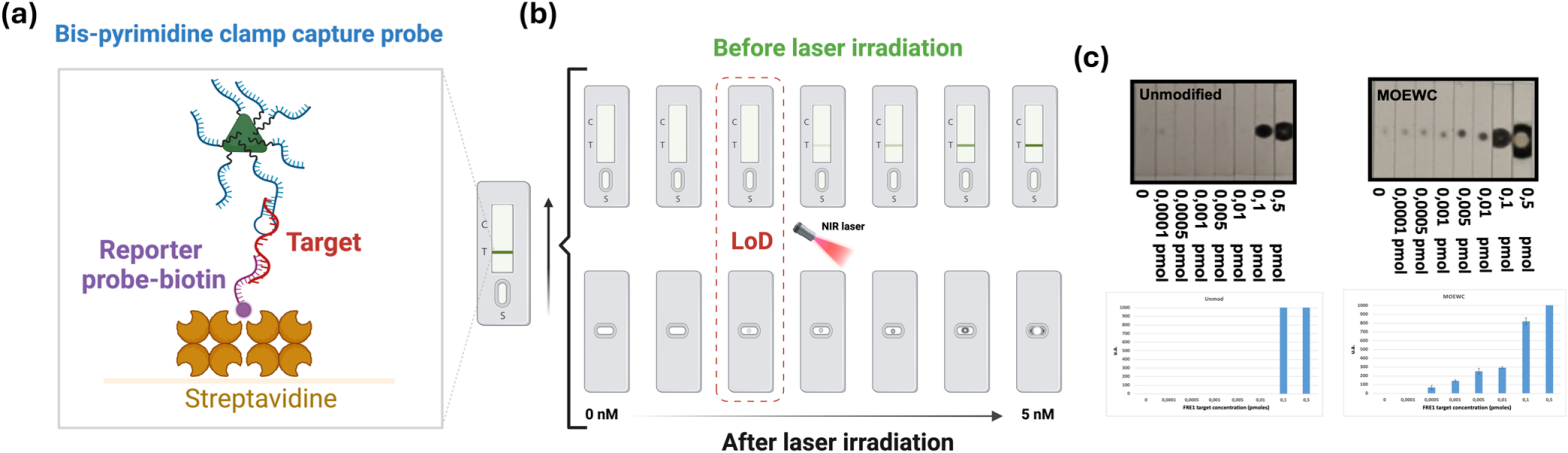
(a) TENADA system representing the trimeric complex formed by the nanoprisms functionalized with the bis-pyrimidine clamp (blue), the reporter biotin probe (purple), and the long synthetic DNA target (red) retained in the lateral flow strip coated with streptavidin. (b) Process used for the determination of the limit of detection (LoD). (c) Results obtained after laser irradiation and quantification of the signal at the backside of the lateral flow strip, comparing Unmodified and MOEWC designs.

The limits of detection (LoD) were determined using synthetic DNA target concentrations ranging from 0 to 5 nM. As shown in Table 3, the modified bis-pyrimidine clamps exhibited LoD values comparable to those of the antiparallel clamps studied in previous works,^7,12^ confirming the suitability of bis-pyrimidine clamps for their use in this system. Notably, the Unmodified bis-pyrimidine clamp showed a significantly higher LoD (1.0 nM) compared to the modified clamps, consistent with the previous biophysical studies (Table 3). In some of the probes, such as the FANA series, we obtained a range in the limit of detection. This variability is due to the high sensitivity of the equipment used to quantify the spots on the thermosensitive paper, which resulted in different values across replicates. To improve accuracy, one possible solution would be to include intermediate points if a more precise measurement is required.

**Table 3:** Limits of Detection Obtained in the Thermal Lateral Flow Sensing Assay for the Unmodified Bis-pyrimidine Clamp and Complementary Sequences Compared to the 2′-FANA and 2′-MOE-Modified Clamps in pmol and nM

name	LoD (pmol)	LoD (nM)
NH_2_-T_5_-Unmodified	0.1	1.0
NH_2_-T_5_-FANAHoog	0.001–0.0005	0.01–0.005
NH_2_-T_5_- FANAWC	0.005–0.001	0.05–0.01
NH_2_-T_5_-FANACompl	0.005–0.001	0.05–0.01
NH_2_-T_5_-MOEHoog	0.0005	0.005
NH_2_-T_5_-MOEWC	0.0001	0.001
NH_2_-T_5_-MOECompl	0.0005	0.005

To investigate the impact of triplex formation relative to duplex-forming oligonucleotides, we synthesized two complementary sequences modified with 2′FANA (FANACompl) and 2′MOE (MOECompl). These complementary sequences showed similar LoD values compared to those of the corresponding modified bis-pyrimidine clamps. Additionally, we performed experiments at pH 6 (data not shown), where the bis-pyrimidine clamps were expected to perform better than the duplex-forming oligonucleotides due to increased triplex stability at lower pH. However, the performance of the clamps did not improve compared to the experiments conducted at pH 7, likely because the Biotin–Streptavidin complex is negatively affected at pH 6.

These results suggest that unlike the antiparallel clamps these modified bis-pyrimidine clamps do not provide a significant stabilization effect under the conditions used for the biosensor experiments when compared to the duplex-forming sequences. We hypothesize that this difference arises from the absence of a preformed structure in the bis-pyrimidine clamp before binding to the target, a feature present in antiparallel clamps. When immobilized on the nanoprism surface, the kinetic advantage of a preformed structure over the duplex-forming sequence may be critical for the recognition and binding to the targeted sequence. Alternative detection systems that do not rely on protein complexes and are compatible with lower pH conditions may benefit from the properties of these modified bis-pyrimidine clamps, potentially achieving sensitivity greater than that of the duplex-forming counterparts.

## 4 Discussion

Triplex formation by synthetic oligonucleotides is an area of great interest due to their potential application in the regulation of gene expression, sensing, and nanotechnology,^8–10^ as demonstrated recently by a multitude of studies exploring these motifs and other noncanonical structures and their ligands.^44^ Although major developments in triplex recognition technologies were made between 1990 and 2010,^9,10^ including peptide nucleic acids (PNA) derivatives,^18,45^ and modified nucleosides that deal with interruptions in the polypurine track,^46^ the major part of diagnostic and therapeutic applications using oligonucleotides are based on duplex formation. The main reasons are the poor binding affinity of unmodified TFOs, the difficulty of finding highly specific long polypurine-polypyrimidine sequences, and the poor solubility of the PNA derivatives. Recently, the development of PPRH technology^47^ and clamp antigene oligonucleotides^48^ has provided a novel perspective for the use of triplexes as therapeutic and biosensing agents, as shown in the development of the TENADA detection method and the antiviral activity of these designs against SARS-CoV-2.^7,12,49^ These clamps can tolerate few interruptions in the targeted polypurine-polypyrimidine tracks, maintaining high stability and expanding the putative triplex-forming sequences that can be targeted.

In this communication, we wanted to explore the possibility of targeting polypurine sequences by using bis-pyrimidine clamps. Due to the poor thermal stability at neutral pH conditions of the parallel triplexes formed by the clamps, we incorporated several modifications. Our studies have shown that chemical modifications at the 2′ position of the sugar moiety can dramatically influence the stability of parallel triplexes. 2′-FANA modifications exhibited the greatest stabilization effect when incorporated into both strands of the clamp. The preference of the sugar puckering for a C2′-endo conformation resulted in favorable arrangement of the three strands that constitute the structure. Other favorable interactions involving 2′-fluorine atoms with neighboring residues could also explain the higher stabilization observed for these sequences, as reported for other fluorinated structures.^38,50^ In contrast, the inversion of the fluorine atom (2′-FRIBO), which shifts the equilibrium to the C3′-endo form, results in the destabilization of the triplex. Interestingly, when these fluorinated modifications were positioned on the Hoogsteen strand (FRIBOHoog), they exerted a negative impact on the stability of the parallel triplex, likely due to the decreased p*K*_a_ of the cytosines induced by the presence of a fluorine atom at the 2′ position.

Surprisingly, although 2′-*O*-alkyl modifications also shift the equilibrium to the less favorable C3′-endo conformation, the hydrophobic effect imparted by 2′-OMe residues on the Hoogsteen strand compensated for the repulsion of the phosphate groups and stabilized the triplexes (OMeHoog). Additionally, although 2′-MOE modifications are probably too large to fit into the major groove of the triplex when present in the Hoogsteen strand, they showed to be stabilizing when positioned on the Watson–Crick strand (MOEWC).

To clarify whether this stabilization effect was due to the presence of 5-methyl-pyrimidines when using 2′-MOE, two additional clamps were synthesized. Only the sequences modified with 5-methyl-pyrimidines in the Hoogsteen sequence imparted a stabilization effect, thanks to the increased basicity of the cytosines, in agreement with previous studies.^25,51^ Therefore, 2′-MOE residues play an important role in triplex stabilization when they are present in the Watson–Crick pyrimidine sequence. To the best of our knowledge, this is the first time that 2′-MOE modifications have been studied in the context of triplex formation. Further studies should be conducted in this direction to understand the superior stabilization conferred by these residues compared to other 2′-*O*-alkyl modifications.

Using clamps for our studies opened the possibility of investigating the effect of modifications on other positions of the triplexes, which is typically not explored when working with TFOs. While many studies have reported the effects of modifications on the pyrimidine strand, which forms the Hoogsteen hydrogen bonds,^25,26,28,52,53^ very few have studied how modifications on the Watson–Crick strand can influence triplex formation.^54^ Indeed, the two most stable parallel triplexes studied (FANAWC and MOEWC) had modifications on one of their Watson–Crick strands. We hypothesize that three main factors play significant roles in this stabilization. First, 2′-sugar and 5-methyl-pyrimidine modifications affect the p*K*_a_ of cytosines, thus, at neutral pH, they can influence the stability of the parallel triplexes when present in the Hoogsteen strand. Second, the conformation of the double helix is crucial for the recognition of the Hoogsteen strand. Modifications that can affect the flexibility, shape, and stability of the double helix are directly correlated with the overall stability of the triplex. Finally, short contacts of the modifications with neighboring residues can stabilize or destabilize the parallel triplexes depending on their spatial disposition within the structure, for instance, the ability of fluorine to form pseudohydrogen bonds could impart an enhanced stabilization effect compared to the other modifications studied. Further structural and computational studies using model sequences could provide insights into these features and explain the striking stabilization effect imparted by certain modifications.

To demonstrate the potential of the most stable bis-pyrimidine clamps as capture probes, we employed a thermal lateral flow device using the TENADA method. These modified bis-pyrimidine clamps reduced the LoD of the unmodified sequence by two to 3 orders of magnitude, achieving affinities comparable to their antiparallel counterparts. However, the modified duplex-forming oligonucleotides (FANACompl and MOECompl) displayed LoD values comparable to those of the bis-pyrimidine clamps, suggesting that the triplex-forming component of the clamps did not provide a significant stabilization advantage under the experimental conditions used. Additional experiments conducted at more acidic pH values did not lead to improved clamp performance, likely due to limitations related to our system when working under these pH conditions.

We hypothesize that the structural characteristics of the capture probe may play a critical role in biosensor sensitivity. In contrast to the bis-pyrimidine clamps, antiparallel clamps (having a preformed structure) demonstrated LoDs two to 3 orders of magnitude lower than those of their duplex-forming counterparts, highlighting the potential importance of structural preorganization in enhancing detection sensitivity.^7,12^ Therefore, although the bis-pyrimidine clamps were shown to be compatible with the thermal lateral flow system studied, the modified complementary sequences and antiparallel clamps would be preferred due to their simplicity. In contrast, in systems capable of operating at lower pH values, we would expect the modified bis-pyrimidine clamps to exhibit enhanced performance.

This work will contribute to expanding our knowledge of the specific interactions that most effectively stabilize the triplexes formed by the bis-pyrimidine clamps and the optimal modification strategies required to enhance the applicability of these oligonucleotides. Notably, this study delves into a context not often explored where more than one pyrimidine interruption is present in the formation of the triplex motif. The incorporation of these pyrimidine interruptions is necessary to increase the specificity of the clamps when targeting genomes with reduced polypurine sequences. However, future research regarding the use of modified nucleosides designed to deal with interruptions may be beneficial.^46^

Furthermore, the implementation of these novel modified bis-pyrimidine clamps has the potential to broaden the targeting scope of the designs, including polypurine sequences. This may lead to an increase in the number of triplex-forming strands available for use in future diagnostic and therapeutic applications.

## 5 Conclusions

We have demonstrated that the incorporation of 2′-sugar and 5-methyl-pyrimidine modifications into both strands of a bis-pyrimidine clamp can enhance its binding affinity by stabilizing the parallel triplex formed. These modifications have effects not only when they are located on the strand directly implicated in the formation of the triplex (Hoogsteen strand) but also when they are present on one of the strands that form the duplex (Watson–Crick strand).

Moreover, the bis-pyrimidine clamps that formed the most stable parallel triplexes exhibited limits of detection (LoD) two to 3 orders of magnitude lower than the unmodified sequence and comparable to previously studied antiparallel clamps in a thermal lateral flow device, highlighting their effectiveness as capture probes.

Apart from broadening the targeting scope of the clamps to include polypurine sequences, the stabilization imparted by certain modifications at neutral pH will enhance the applicability of these oligonucleotides as biosensing agents as well as their ability to target endogenous sequences for therapeutic purposes. Furthermore, the stabilization provided by the fluorine modifications can be used for the study of cellular processes and serve as molecular probes of triplex formation *in cell* using ^13^F NMR experiments.

## Supplementary Material



## Notes

Sequences and schematic representations of the clamp designs studied in this work, melting temperatures from the preoptimization experiments (Section 3.1), plots representing melting experiments, CD, PAGE, NMR, and pH titrations for the bis-pyrimidine clamps not shown in the main text, and raw data from the thermal lateral flow sensing device experiments (PDF)
