# Supplementary material for: Modified Bis-pyrimidine Clamps for Triplex Formation and Their Use in SARS-CoV‑2 Detection
Source: ACS Omega. 2025 May 23;10(22):23535–48. doi: 10.1021/acsomega.5c02155 (PMC12163785; doi:10.1021/acsomega.5c02155)

Supporting information

Modified bis-pyrimidine clamps for triplex formation and their use in SARS-CoV-2 detection

Arnau Domínguez,[1,2,3] Raimundo Gargallo,[4] Carlos Cuestas-Ayllón,[2,5] Irene Gomez-Pinto,[6] Carme Fàbrega,[1,2] Jesús Martínez de la Fuente,[2,5] Masad J. Damha,[7] Carlos González,[6] Ramon Eritja,[*,1,2] and Anna Aviñó[*,1,2]

[1]Instituto de Quimica Avanzada de Cataluña (IQAC), Consejo Superior de Investigaciones Científicas (CSIC), Jordi Girona 18-26, 08034 Barcelona, Spain.

[2]Centro de Investigación Biomédica en Red de Bioingeniería, Biomateriales y Nanomedicina (CIBER-BBN), Madrid, Spain.

[3]Department of Inorganic and Organic Chemistry, University of Barcelona (UB), 08028, Barcelona, Spain.

[4]Department of Chemical Engineering and Analytical Chemistry, University of Barcelona (UB), 08028 Barcelona, Spain.

[5]Instituto de Nanociencia y Materiales de Aragón (INMA), Consejo Superior de Investigaciones Científicas (CSIC), 50018 Zaragoza, Spain.

[6]Instituto de Química Física Blas Cabrera, CSIC, Serrano 119, 28006 Madrid, Spain.

[7]Department of Chemistry, McGill University, Montreal, Quebec H3A 0B8, Canada.

E-mail address: recgma@cid.csic.es (R. Eritja), anna.avinyo@iqac.csic.es (A. Aviñó).

Supplementary tables

Table S1. Sequences used in this work

Table S2. Melting temperatures of bis-pyrimidine clamps with varying number of purine interruptions

Supplementary figures

Fig. S1. Sequence and normalized UV-melting curves of bis-pyrimidine clamps with varying number of purine interruptions and the polypurine target

Fig. S2. Scheme of the bis-pyrimidine clamps studied in this work.

Fig. S3. Normalized UV-melting curves of modified bis-pyrimidine clamps

Fig. S4. CD-spectra of Complementary and Control sequences with 5-methyl-modified clamps

Fig. S5. 12 % native PAGE of 2'-*O*-alkyl modified bis-pyrimidine clamps

Fig. S6. ^{1}H NMR melting experiments of the exchangeable imino region of the studied bis-pyrimidine clamps between 5 and 45 ºC.

Fig. S7. Univariate analysis of the pH-titrations' spectra with the corresponding fitted sigmoidal curves.

Fig. S8. UV-spectra of Unmodified, ControlHoog and 5-Me-dC clamps recorded during the pH-titrations and fitted data using the univariate analysis.

Fig. S9. Concentration profiles and pure spectra of 2'-*O*-alkyl modified clamps obtained from the multivariate analysis.

Fig. S10. Concentration profiles and pure spectra of Unmodified, ControlHoog and 5-Me-dC clamps obtained from the multivariate analysis.

Supplementary tables

Table S1. Sequences used in this work.

Name	Sequence (5'-3')
**Unmodified**	CCT TCT TCT TCA TCC TCA TCT TTT TTC TAC TCC TAC TTC TTC TTC C
**Complementary**	CCT TCT TCT TCA TCC TCA TCT
**Control**	CCT TCT TCT TCA TCC TCA TCT TTT TTA TCT TCA CCT CTT CCT TCC T
**Target**	AGA TGA GGA TGA AGA AGA AGG TGA
**MOEHoog**	CCT TCT TCT TCA TCC TCA TCT TTT T(moT)(moC) (moT)(moA)(moC) (moT)(moC)(moC) (moT)(moA)(moC) (moT)(moT)(moC) (moT)(moT)(moC) (moT)(moT)(moC) (moC)
**MOEWC**	(moC)(moC)(moT) (moT)(moC)(moT) (moT)(moC)(moT) (moT)(moC)(moA) (moT)(moC)(moC) (moT)(moC)(moA) (moT)(moC)(moT) TTT TTC TAC TCC TAC TTC TTC TTC C
**OMeHoog**	CCT TCT TCT TCA TCC TCA TCT TTT T(meU)(meC) (meU)(meA)(meC) (meU)(meC)(meC) (meU)(meA)(meC) (meU)(meU)(meC) (meU)(meU)(meC) (meU)(meU)(meC) (meC)
**OMeWC**	(meC)(meC)(meU) (meU)(meC)(meU) (meU)(meC)(meU) (meU)(meC)(meA) (meU)(meC)(meC) (meU)(meC)(meA) (meU)(meC)(meU) TTT TTC TAC TCC TAC TTC TTC TTC CAC T
**5MedCHoog**	CCT TCT TCT TCA TCC TCA TCT TTT TT(5mC) TA(5mC) T(5mC)(5mC) TA(5mC) TT(5mC) TT(5mC) TT(5mC) (5mC)
**5MedCWC**	(5mC)(5mC)T T(5mC)T T(5mC)T T(5mC)A T(5mC)(5mC) T(5mC)A T(5mC)T TTT TTC TAC TCC TAC TTC TTC TTC C
**FANAHoog**	CCT TCT TCT TCA TCC TCA TCT TTT T(afT)(afC) (afT)(afA)(afC) (afT)(afC)(afC) (afT)(afA)(afC) (afT)(afT)(afC) (afT)(afT)(afC) (afT)(afT)(afC) (afC)
**FANAWC**	(afC)(afC)(afT) (afT)(afC)(afT) (afT)(afC)(afT) (afT)(afC)(afA) (afT)(afC)(afC) (afT)(afC)(afA) (afT)(afC)(afT) TTT TTC TAC TCC TAC TTC TTC TTC C
**FRIBOHoog**	CCT TCT TCT TCA TCC TCA TCT TTT T(rfU)(rfC) (rfU)(rfA)(rfC) (rfU)(rfC)(rfC) (rfU)(rfA)(rfC) (rfU)(rfU)(rfC) (rfU)(rfU)(rfC) (rfU)(rfU)(rfC) (rfC)
**FRIBOWC**	(rfC)(rfC)(rfU) (rfU)(rfC)(rfU) (rfU)(rfC)(rfU) (rfU)(rfC)(rfA) (rfU)(rfC)(rfC) (rfU)(rfC)(rfA) (rfU)(rfC)(rfU) TTT TTC TAC TCC TAC TTC TTC TTC CAC T

moT: 5-methyl-2'-MOE-U, moC: 5-methyl-2'-MOE-C, moA: 2'-MOE-A, meU: 2'-O-methyl-U, meC: 2'-O-methyl-C, meA: 2'-O-methyl-A, 5mC: 5-methyl-C, afT: 2'-F-araT, afC: 2'-F-araC, afA: 2'-F-araA, rfU: 2'-F-U, rfC: 2'-F-riboC, rfA: 2'-F-riboA.

Table S2. UV melting curves of the 1:1 mixture of the bis-pyrimidine clamps with the polypurine target at pH 5.0, 6.0, and 7.0 monitored at 260 nm. pH 5.0: 100 mM sodium phosphate/citrate buffer and 100 mM NaCl. pH 6.0,7.0: 100 mM sodium phosphate and 100 mM NaCl. The oligonucleotide concentration was 2 µM.

Number of mismatches	T_m Triplex (ºC)		T_m Duplex (ºC)		
	pH 6.0	pH 7.0	pH 5.0[a]	pH 6.0	pH 7.0
3	40.4 ± 0.2	9.0 ± 1.0	66.4 ± 0.5	68.5 ± 0.2	73.2 ± 0.9
2	44.0 ± 0.4	11.4 ± 0.1	65.2 ± 0.3	64.7 ± 0.2	62.2 ± 0.2
1[b]	n.d	n.d	37.9 ± 0.6	32.7 ± 0.4	33.0 ± 0.3

[a] Single transition observed at pH 5.0. [b] pH-independent transition. n.d: Not detected.

Table S3. Sequences used in the thermal lateral flow assay. moT: 5-methyl-2'-MOE-U, moC: 5-methyl-2'-MOE-C, moA: 2'-MOE-A, afT: 2'-F-araT, afC: 2'-F-araC, afA: 2'-F-araA.

Name	Sequence (5'-3')
**LongTarget Reporter-Biotin**	ATGTATTGTTCTTTCTACCCTCCAGATGAGGATGAAGAAGAAGGTGA GGGTAGAAAGAACAATACAT-Biotin
**NH_2-T_5-Unmodified**	NH_2-TTT TTC CTT CTT CTT CAT CCT CAT CTT TTT TCT ACT CCT ACT TCT TCT TCC
**NH_2-T_5-Control**	NH_2-TTT TTC CTT CTT CTT CAT CCT CAT CTT TTT TAT CTT CAC CTC TTC CTT CCT
**NH_2-T_5-FANAHoog**	NH_2-TTT TTC CTT CTT CTT CAT CCT CAT CTT TTT (afT)(afC) (afT) (afA)(afC)(afT) (afC)(afC)(afT) (afA)(afC)(afT) (afT)(afC)(afT) (afT)(afC)(afT) (afT)(afC)(afC)
**NH_2-T_5- FANAWC**	NH_2-TTT TT(afC) (afC)(afT)(afT) (afC)(afT)(afT) (afC)(afT)(afT) (afC)(afA)(afT) (afC)(afC)(afT) (afC)(afA)(afT) (afC)(afT)T TTT TCT ACT CCT ACT TCT TCT TCC
**NH_2-T_5-FANACompl**	(afC)(afC)(afT) (afT)(afC)(afT) (afT)(afC)(afT) (afT)(afC)(afA) (afT)(afC)(afC) (afT)(afC)(afA) (afT)(afC)(afT)
**NH_2-T_5-MOEHoog**	NH_2-TTT TTC CTT CTT CTT CAT CCT CAT CTT TTT (moT)(moC)(moT) (moA)(moC)(moT) (moC)(moC)(moT) (moA)(moC)(moT) (moT)(moC)(moT) (moT)(moC)(moT) (moT)(moC) (moC)
**NH_2-T_5-MOEWC**	NH_2-TTT TT(moC) (moC)(moT)(moT) (moC)(moT)(moT) (moC)(moT)(moT) (moC)(moA)(moT) (moC)(moC)(moT) (moC)(moA)(moT) (moC)(moT)T TTT TCT ACT CCT ACT TCT TCT TCC
**NH_2-T_5-MOECompl**	NH_2-TTT TT(moC) (moC)(moT)(moT) (moC)(moT)(moT) (moC)(moT)(moT) (moC)(moA)(moT) (moC)(moC)(moT) (moC)(moA)(moT) (moC)(moT)

moT: 5-methyl-2'-MOE-U, moC: 5-methyl-2'-MOE-C, moA: 2'-MOE-A, afT: 2'-F-araT, afC: 2'-F-araC, afA: 2'-F-araA.

Supplementary figures

Fig. S1. a) Sequences of the bis-pyrimidine clamps studied with different number of pyrimidine interruptions and the polypurine target. Pyrimidine Watson-Crick, purine Watson-Crick and Hoogsteen or reverse-Hoogsteen strands are represented in blue, red and green boxes, respectively. **b)** Normalized UV melting curves obtained for the bis-pyrimidine clamps with different number of mismatches at pH 5.0, 6.0 and 7.0. pH 5: 100 mM sodium phosphate/citrate buffer and 100 mM NaCl. pH 6.0 ,7.0: 100 mM sodium phosphate and 100 mM NaCl. The oligonucleotide concentration was 2 µM.

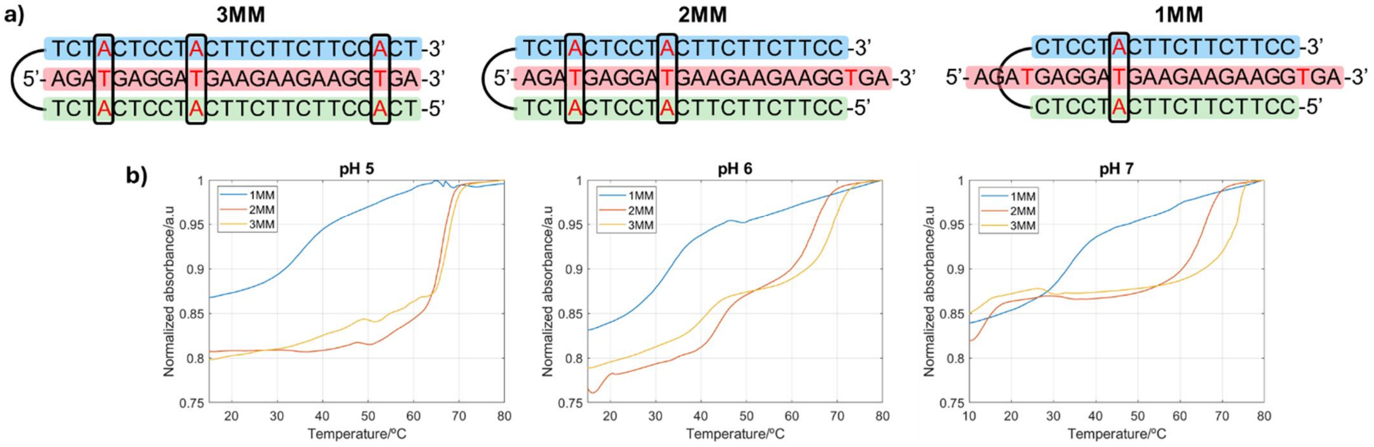

Fig. S2. Scheme of the bis-pyrimidine clamps studied in this work. Hoogsteen pyrimidine strand is depicted as a blue box, the Watson-Crick purine target strand as a red box, and the Watson-Crick pyrimidine strand as a green box. Modifications incorporated into each strand of the clamps are depicted as light blue or light green boxes. In the Control clamp, the label "DNA rand" inside the blue box represents a DNA strand with a randomized sequence that cannot form Hoogsteen hydrogen bonds.

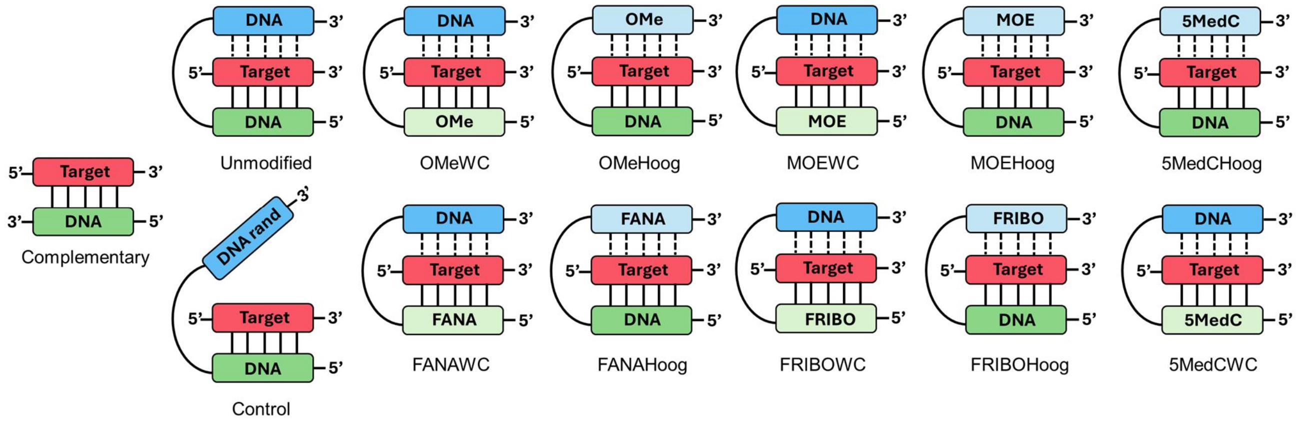

Fig. S3. Normalized UV melting curves obtained for the **a)** 2'-*O*-alkyl modified and **b)** 2'-fluorinated clamps at pH 5.0,6.0 and 7.0. pH 5.0: 100 mM sodium phosphate/citrate buffer and 100 mM NaCl. pH 6.0, 7.0: 100 mM sodium phosphate and 100 mM NaCl. The oligonucleotide concentration was 2 µM.

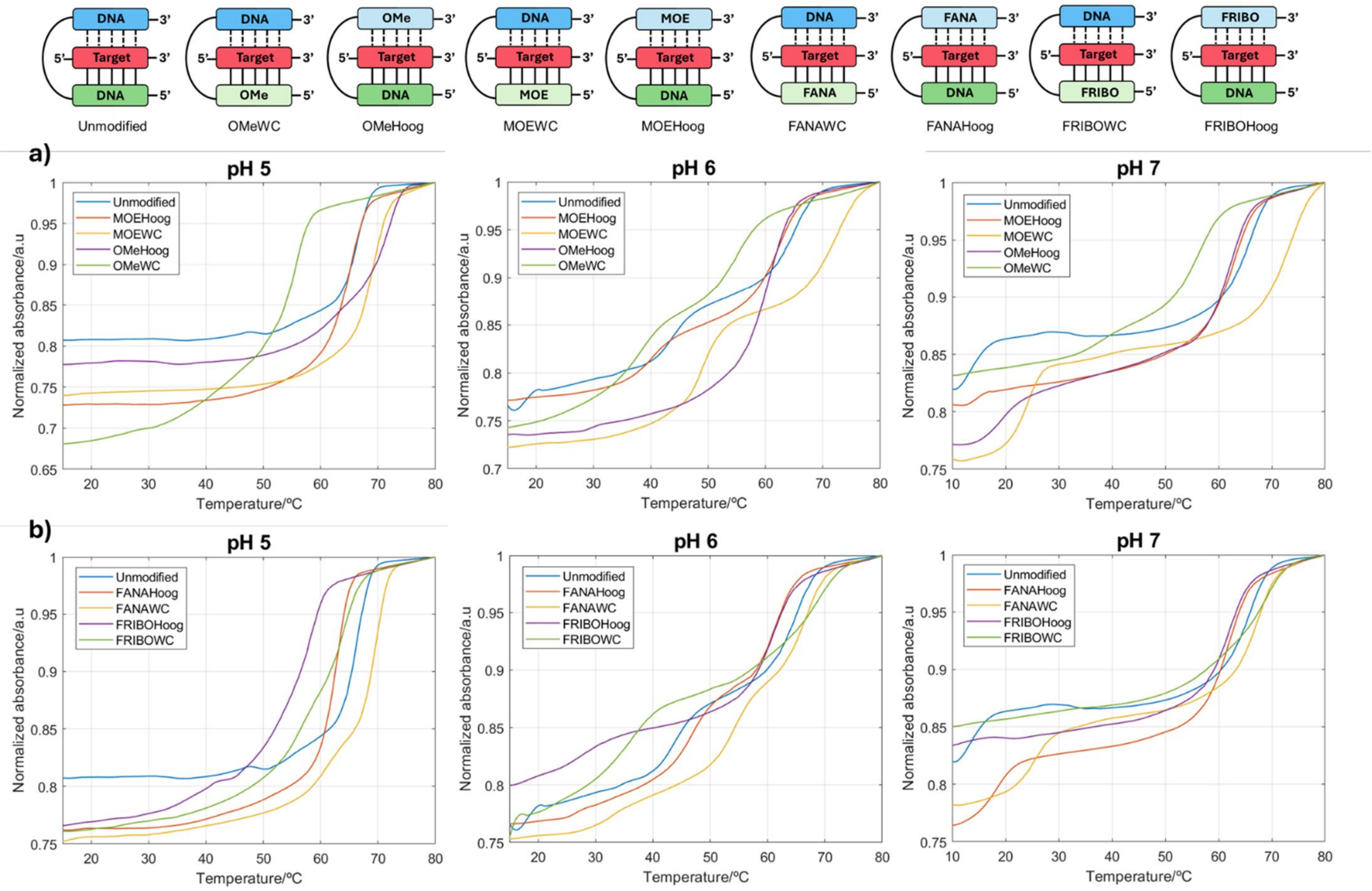

Fig. S4. Circular dichroism spectra recorded between 320-200 nm of the Complementary, Control, and 5-methyl-pyrimidine modified clamps at pH 5.0, 6.0, 7.0 and 10 ºC. pH 5: 100 mM sodium phosphate/citrate buffer and 100 mM NaCl. pH 6.0, 7.0: 100 mM sodium phosphate and 100 mM NaCl. The oligonucleotide concentration was 2 µM.

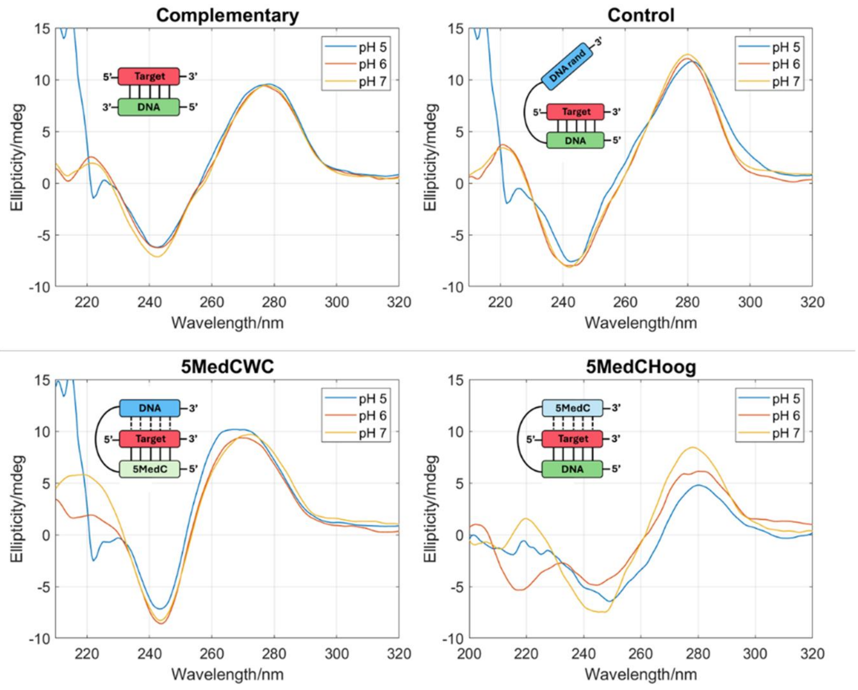

Fig. S5. Native polyacrylamide gel electrophoresis (12 %) comparing Unmodified, Complementary, Control, and 2'-*O*-alkyl modified clamps with and without the addition of the polypurine target. 100 mM sodium phosphate buffer pH 6.0 and 100 mM NaCl. Gels were run at 10 ºC. The oligonucleotide concentration was 2 µM.

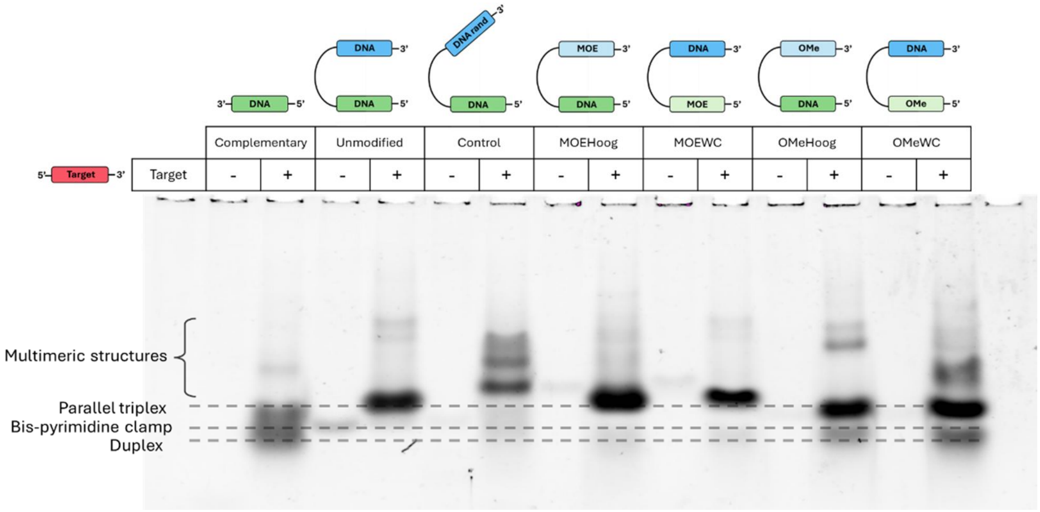

Fig. S6. ^{1}H NMR melting experiments of the exchangeable imino region of the studied bis-pyrimidine clamps at pH 5.0, 6.0, 7.0 and T = 5 ºC. 9:1 H_2O/D_2O in 100 mM sodium phosphate buffer pH 5.0, 6.0 or 7.0 and 100 mM NaCl. The oligonucleotide concentration was 100 µM.

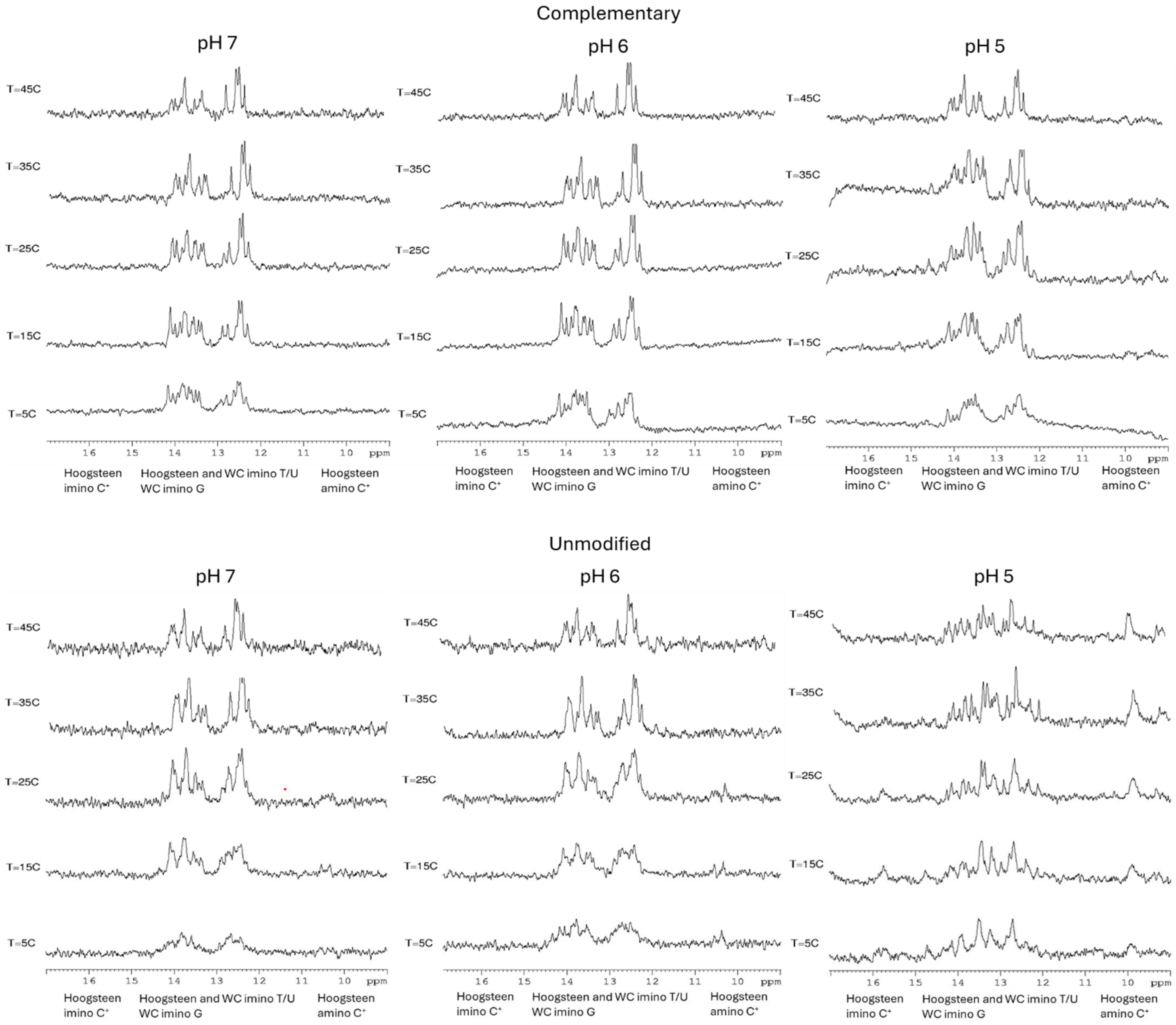

MOEHoog

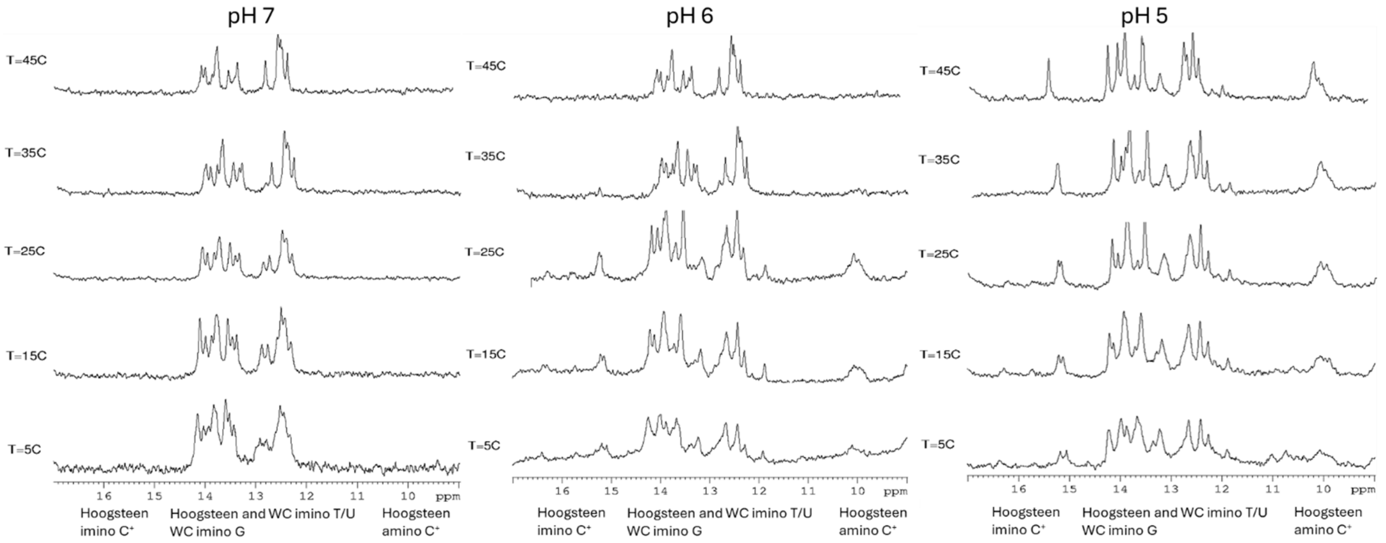

MOEWC

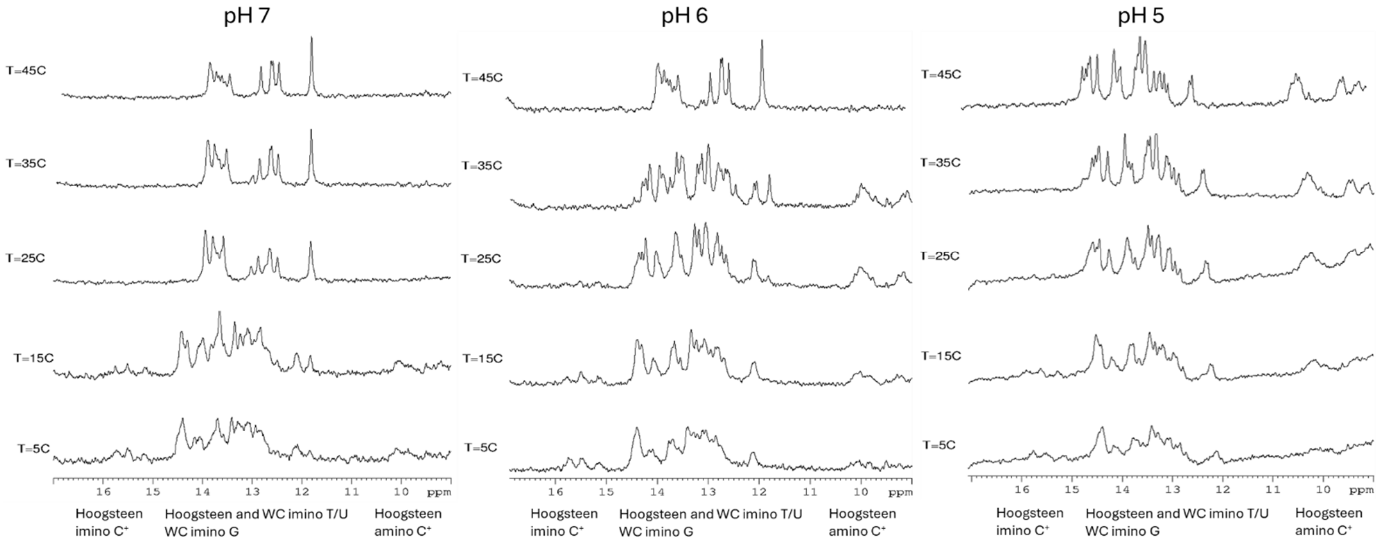

OMeHoog

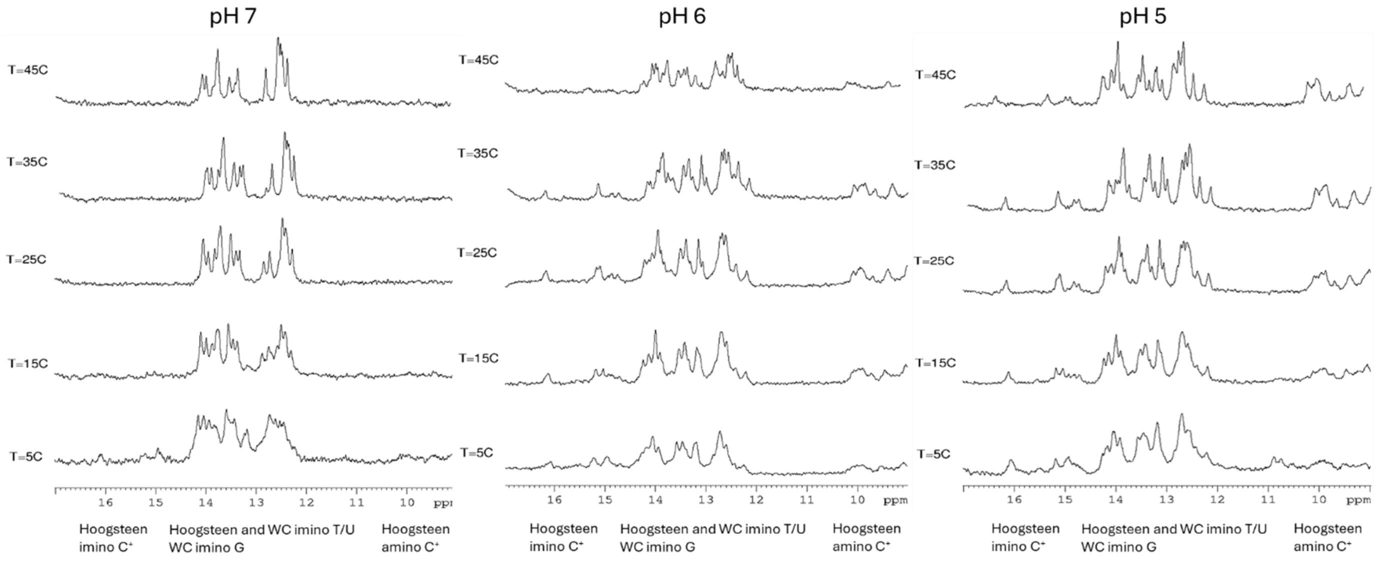

OMeWC

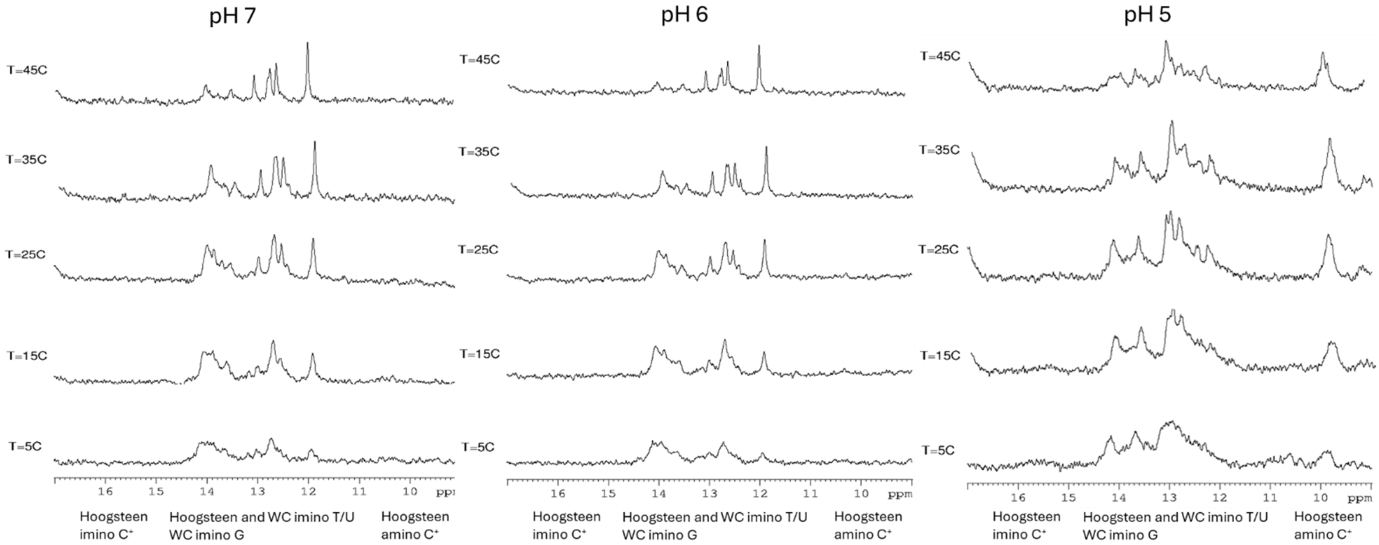

FANAHoog

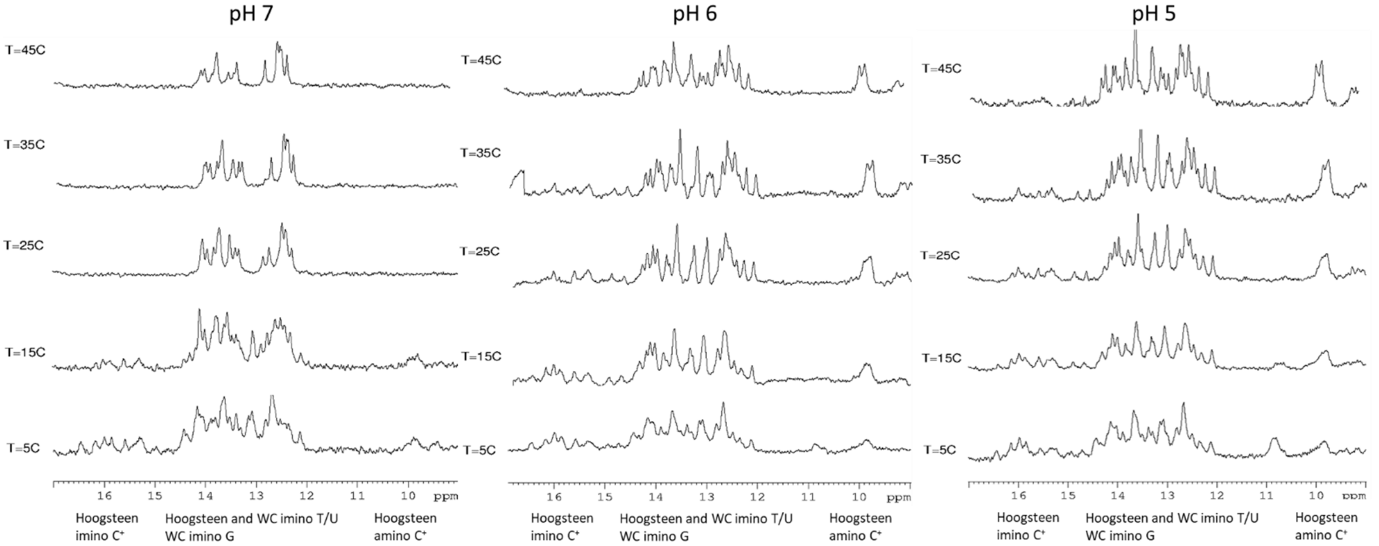

FRIBOHoog

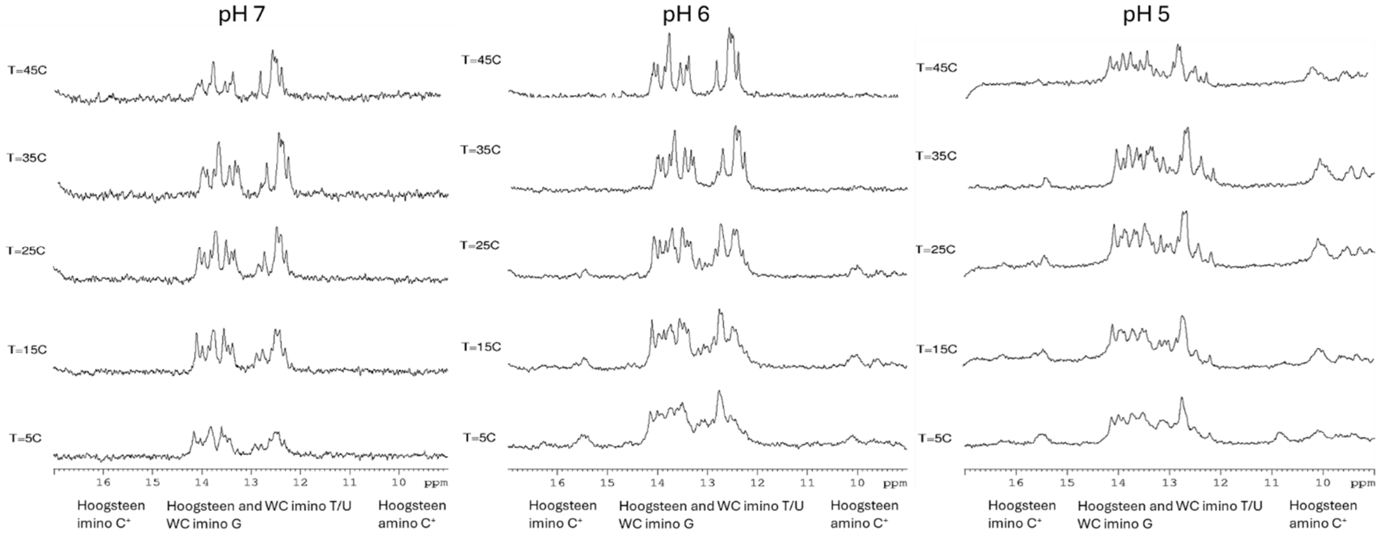

FRIBOWC

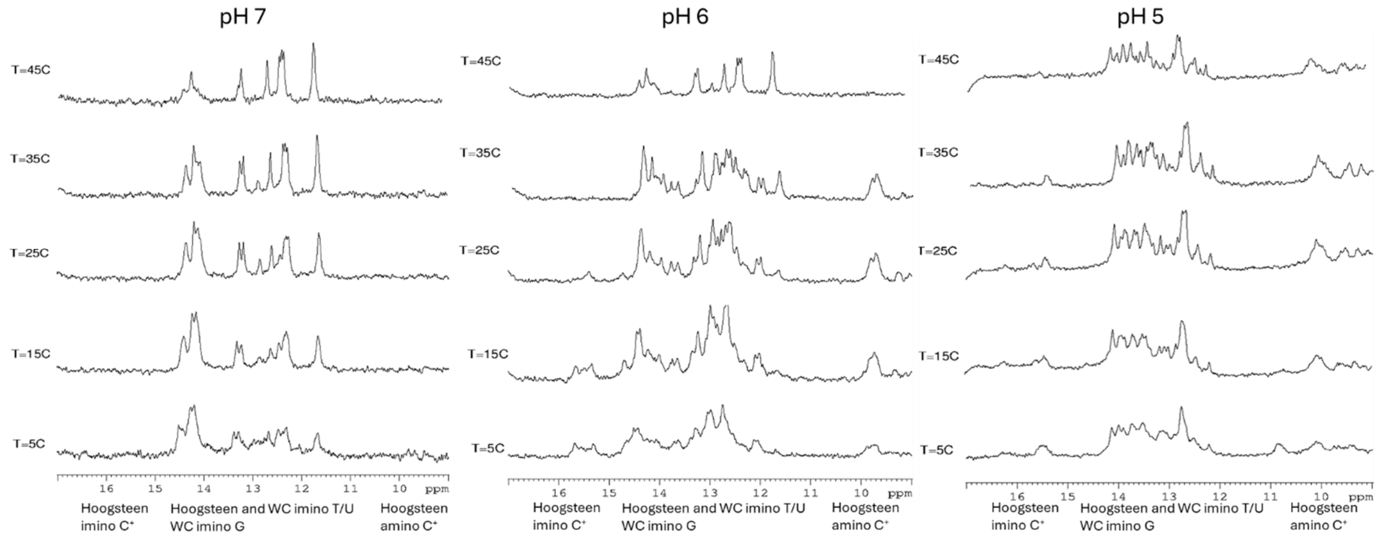

5MedCHoog

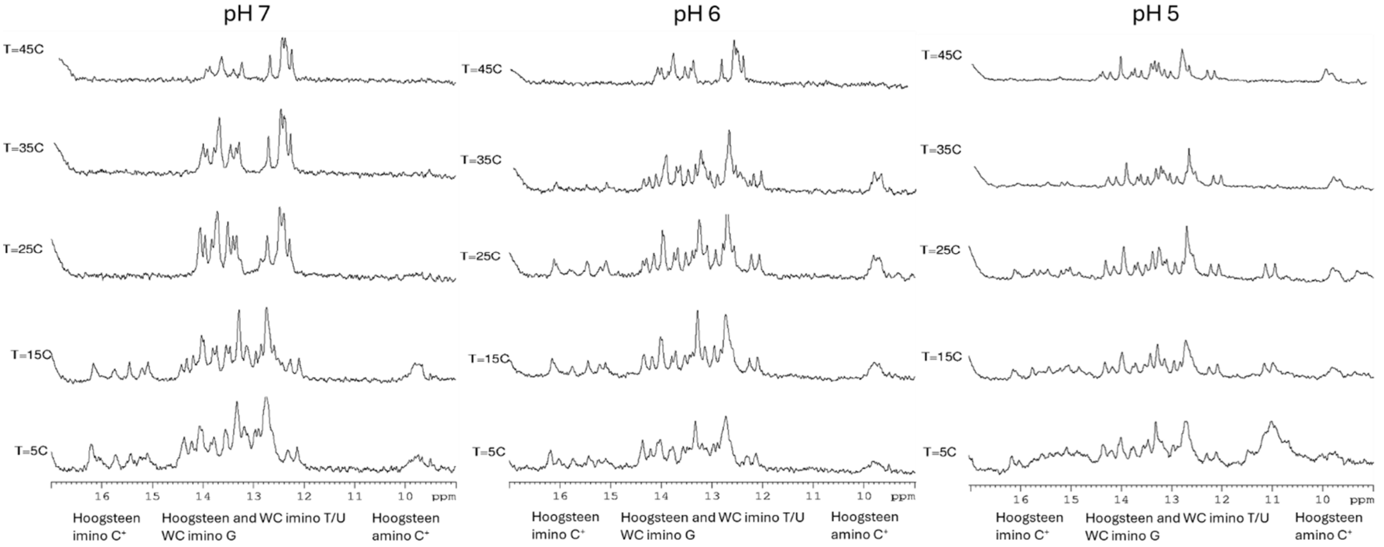

Fig. S7. Univariate analysis of the pH-titrations' spectra with the corresponding fitted sigmoidal curves. **a)** 2'-fluorinated series. **b)** 2'-O-alkyl series. 100 mM Robinson-Britton buffer and 100 mM NaCl. The oligonucleotide concentration was 2 µM.

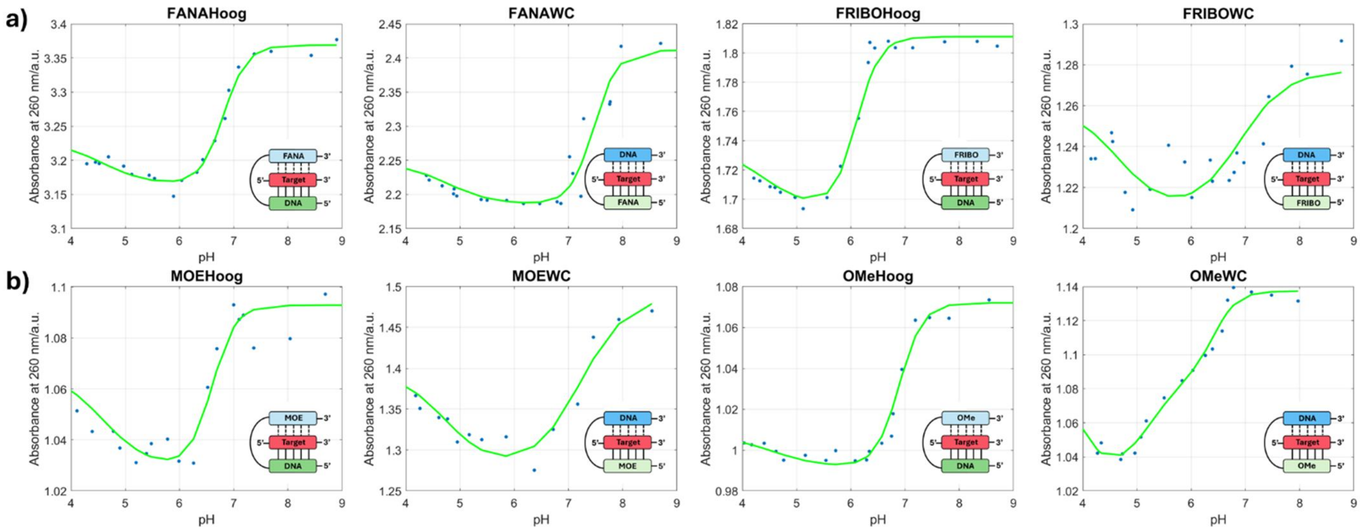

Fig. S8. a) UV spectra of the Unmodified, ControlHoog and 5-Me-dC clamps recorded between 320-220 nm during pH-titrations. **b)** Univariate analysis with the corresponding fitted sigmoidal curves. The buffer was 100 mM Robinson-Britton with 100 mM NaCl. The oligonucleotide concentration was 2 µM.

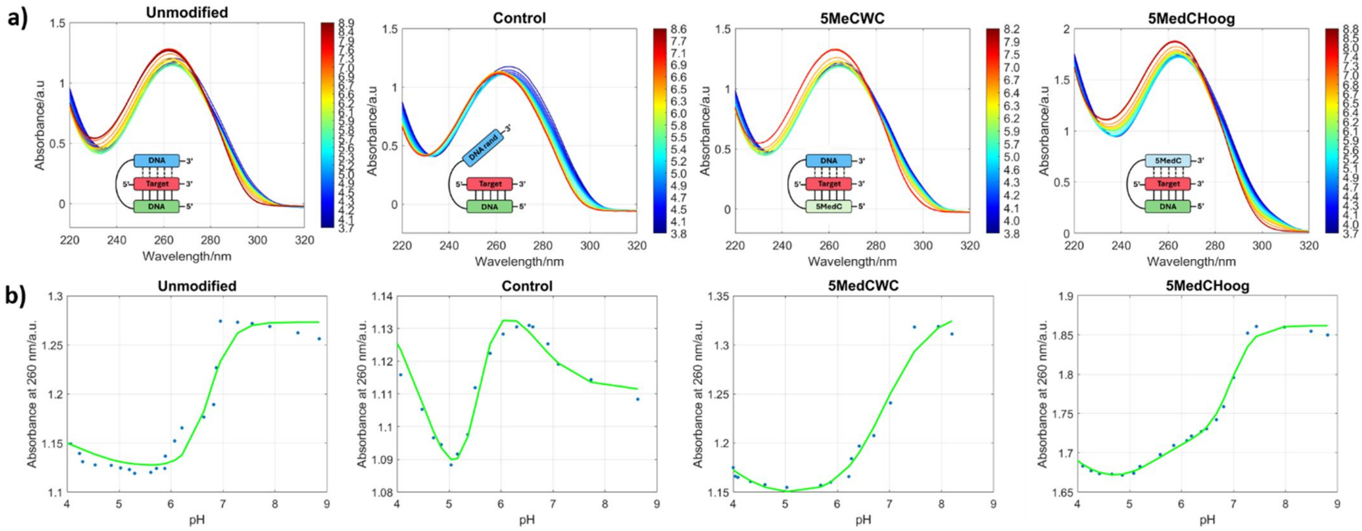

Fig. S9. a) Concentration profiles obtained from the multivariate analysis of the 2'-*O*-alkyl clamps. **b)** pure spectra of the species. Protonated structure, parallel triplex, and triplex unfolded are represented respectively yellow, red, and blue, respectively. OMeWC presents an equilibrium between two species between 4.0 and 6.5 pH values.

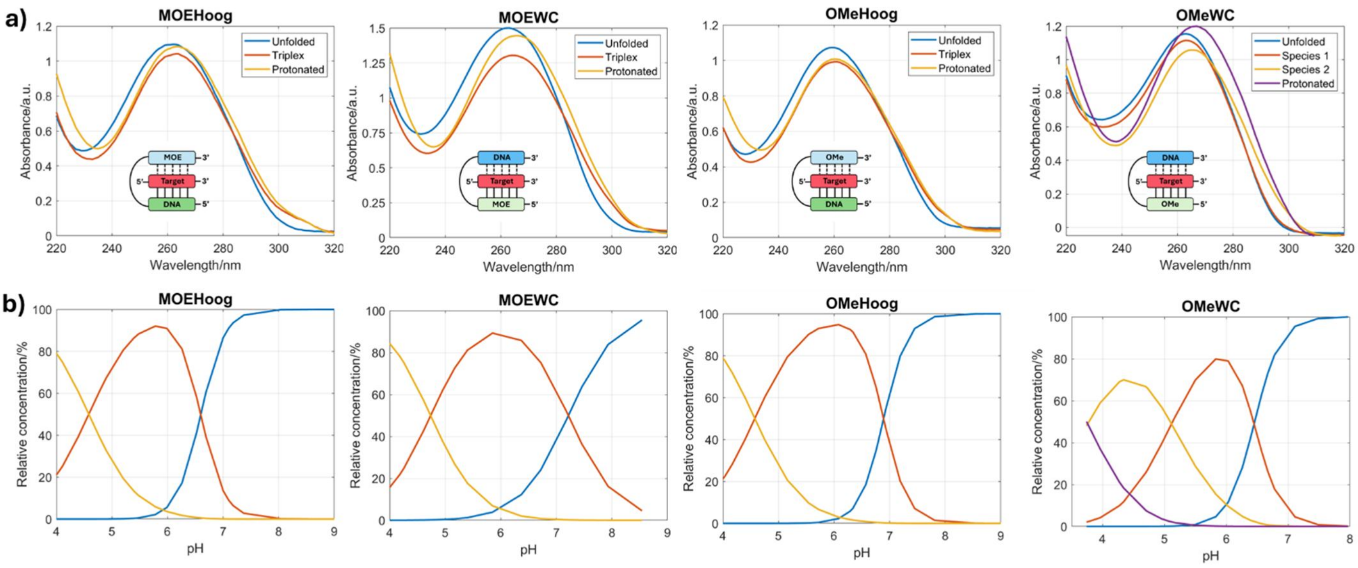

Fig. S10. a) Concentration profiles obtained from the multivariate analysis of the Unmodified, ControlHoog and 5-Me-dC clamps. **b)** pure spectra of the species. Protonated structure, parallel triplex, and triplex unfolded are represented respectively yellow, red, and blue. ControlHoog and 5MedCHoog present more than three species.

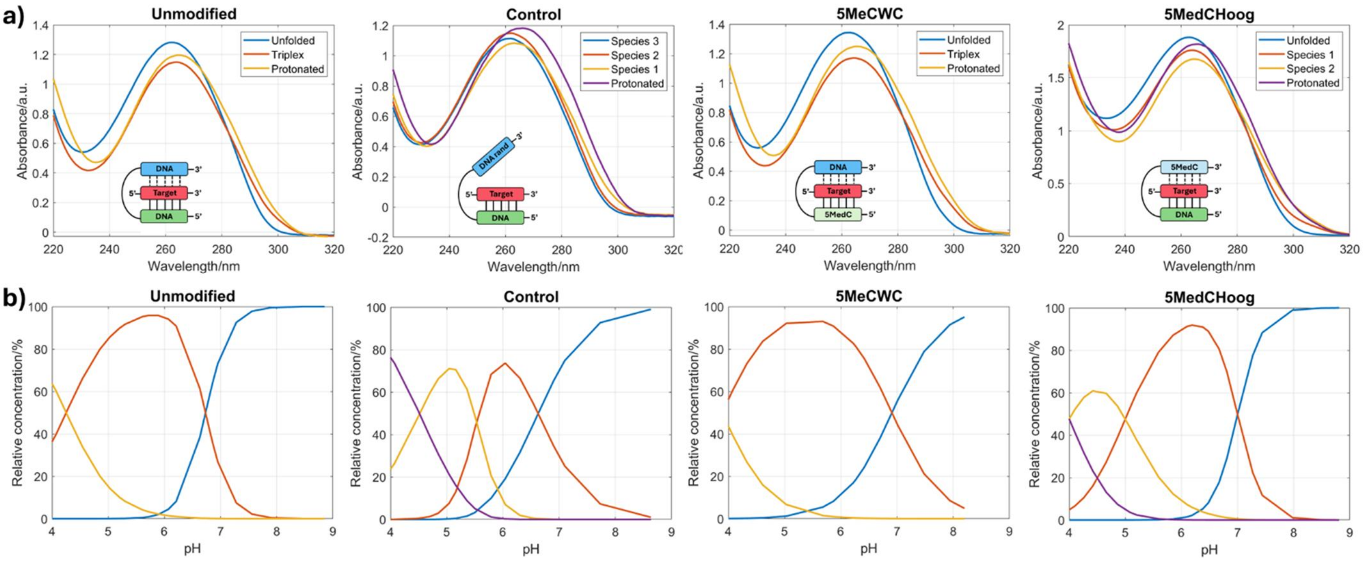

Fig. S11. Results obtained after laser irradiation and the correlation obtained after quantification the spot obtained at the back part of the lateral flow strip to determine the limits of detection with the thermal lateral flow sensing assay for the Unmodified bis-pyrimidine clamp and complementary sequences compared to the 2'-FANA and 2'-MOE modified clamps.

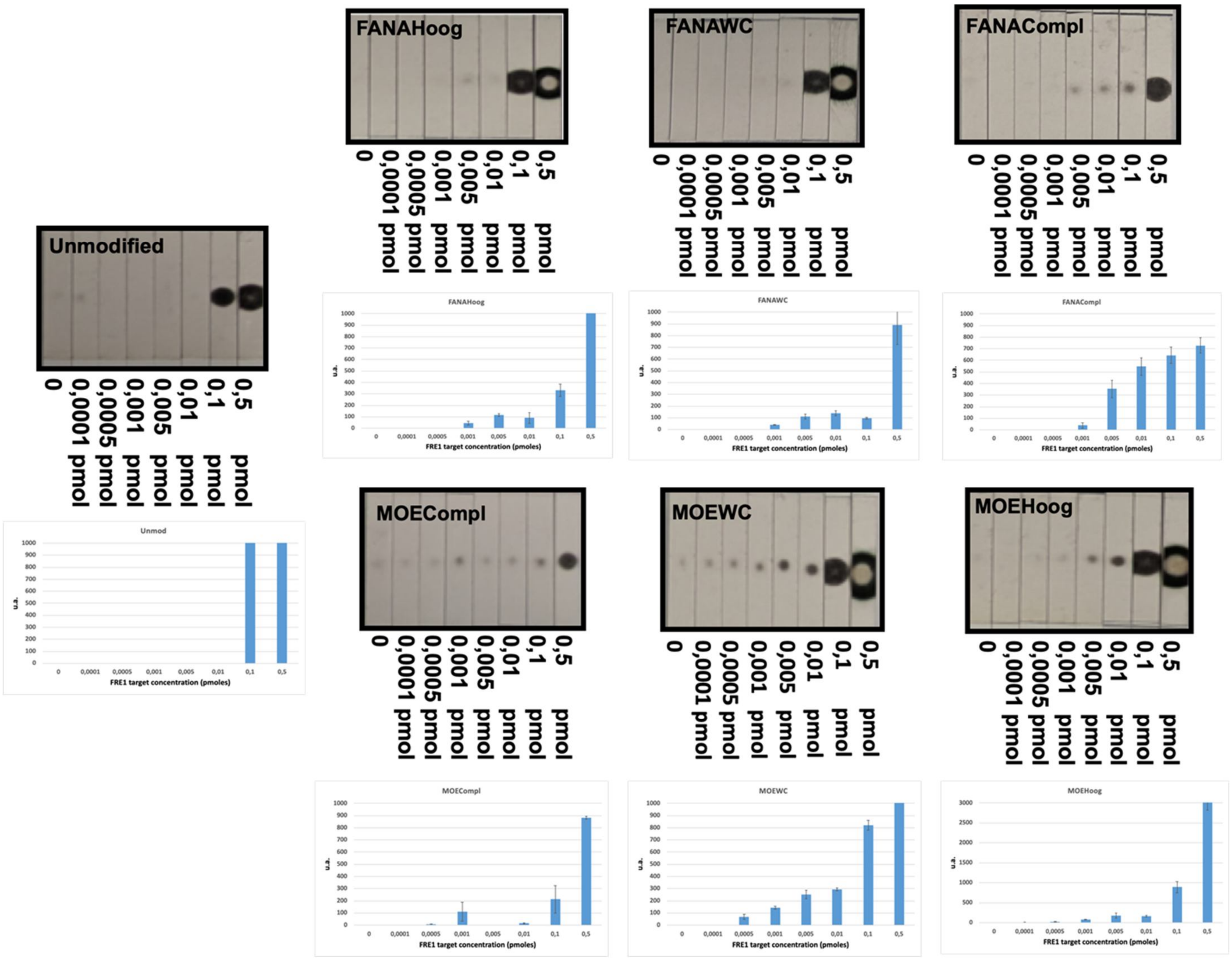

Supplement: Supplementary file 1 [file ao5c02155_si_001.pdf]
